# Interaction of Yna1 and Yna2 Is Required for Nuclear Accumulation and Transcriptional Activation of the Nitrate Assimilation Pathway in the Yeast *Hansenula polymorpha*

**DOI:** 10.1371/journal.pone.0135416

**Published:** 2015-09-03

**Authors:** Lucia Silvestrini, Beatrice Rossi, Andreas Gallmetzer, Martine Mathieu, Claudio Scazzocchio, Enrico Berardi, Joseph Strauss

**Affiliations:** 1 Fungal Genetics and Genomics Unit, Division of Microbial Genetics and Pathogen Interactions, BOKU-University of Natural Resources and Life Sciences Vienna, University and Research Center Tulln, Konrad Lorenz Strasse 24, 3430, Tulln/Donau, Austria; 2 Laboratorio di Genetica Microbica, DiSA, Universitá Politecnica delle Marche, via Brecce Bianche, 60131, Ancona, Italy; 3 Institute for Integrative Biology of the Cell (I2BC), CEA, CNRS, Universitè Paris-Sud, Orsay, France; 4 Department of Microbiology, Imperial College, London, United Kingdom; 5 Health and Environment Department, Austrian Institute of Technology GmbH (AIT), University and Research Center Tulln, Konrad Lorenz Strasse 24, 3430, Tulln/Donau, Austria; University of Nebraska-Lincoln, UNITED STATES

## Abstract

A few yeasts, including *Hansenula polymorpha* are able to assimilate nitrate and use it as nitrogen source. The genes necessary for nitrate assimilation are organised in this organism as a cluster comprising those encoding nitrate reductase *(YNR1)*, nitrite reductase *(YNI1)*, a high affinity transporter *(YNT1)*, as well as the two pathway specific Zn(II)_2_Cys_2_ transcriptional activators *(YNA1*, *YNA2)*. Yna1p and Yna2p mediate induction of the system and here we show that their functions are interdependent. Yna1p activates *YNA2* as well as its own *(YNA1)* transcription thus forming a nitrate-dependent autoactivation loop. Using a split-YFP approach we demonstrate here that Yna1p and Yna2p form a heterodimer independently of the inducer and despite both Yna1p and Yna2p can occupy the target promoter as mono- or homodimer individually, these proteins are transcriptionally incompetent. Subsequently, the transcription factors target genes containing a conserved DNA motif (termed nitrate-UAS) determined in this work by *in vitro* and *in vivo* protein-DNA interaction studies. These events lead to a rearrangement of the chromatin landscape on the target promoters and are associated with the onset of transcription of these target genes. In contrast to other fungi and plants, in which nuclear accumulation of the pathway-specific transcription factors only occur in the presence of nitrate, Yna1p and Yna2p are constitutively nuclear in *H*. *polymorpha*. Yna2p is needed for this nuclear accumulation and Yna1p is incapable of strictly positioning in the nucleus without Yna2p. *In vivo* DNA footprinting and ChIP analyses revealed that the permanently nuclear Yna1p/Yna2p heterodimer only binds to the nitrate-UAS when the inducer is present. The nitrate-dependent up-regulation of one partner protein in the heterodimeric complex is functionally similar to the nitrate-dependent activation of nuclear accumulation in other systems.

## Introduction

**Fig 1:**
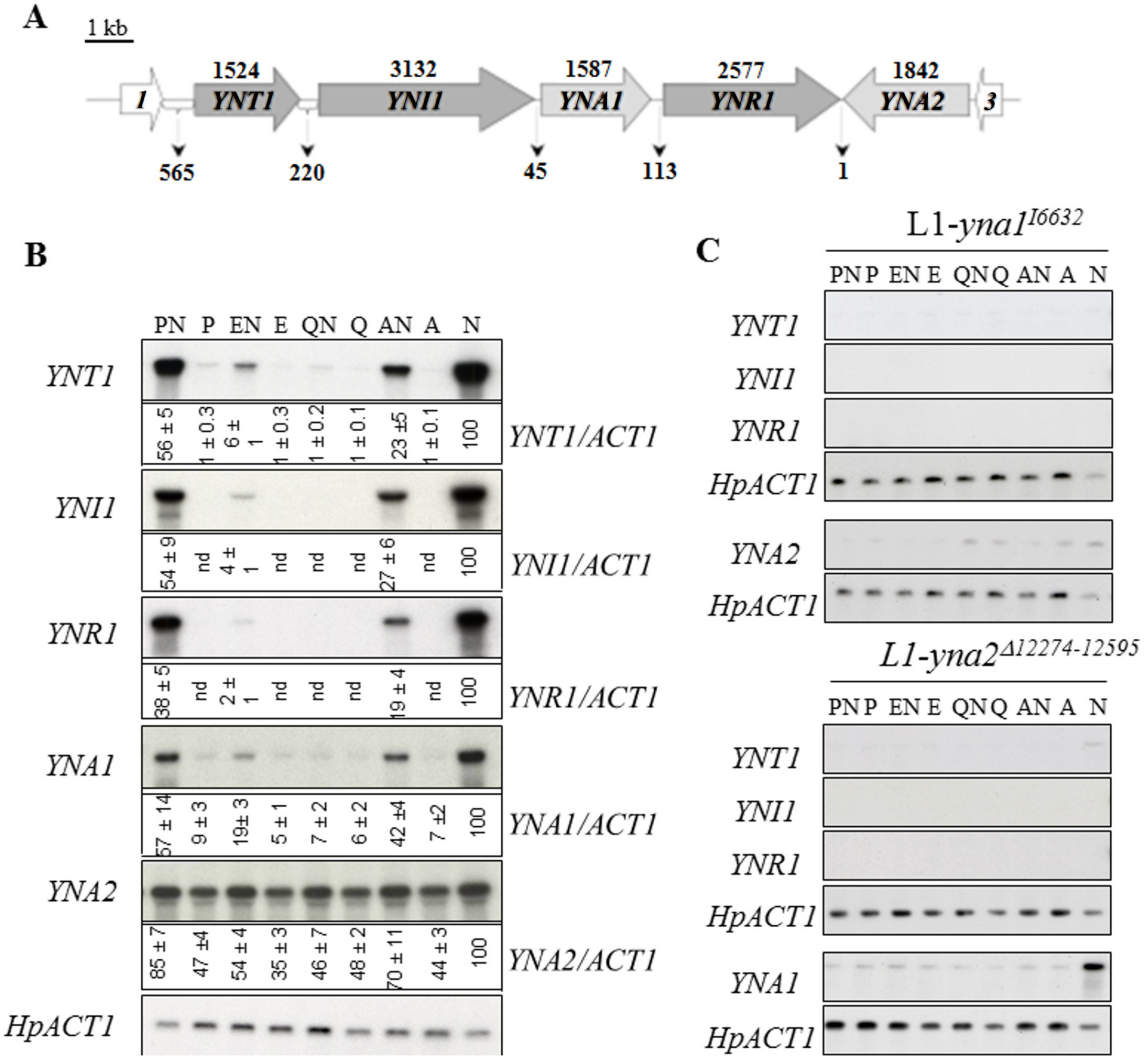
The *H*. *polymorpha* nitrate utilization cluster (GenBank #AJ223294) and its regulation. **A.** The complete sequence (nt 1–13684) consists of five nitrate assimilation genes (grey shaded) flanked by a predicted glutathione S-transferase (*ORF-1)* on the one side and by a predicted *RAD3* homolog (*ORF-3*) on the other. Numbers (in bp) above the coding regions correspond to the size of the ORFs and numbers between the genes indicate the length of the intergenic regions. *YNT1*, nitrate transporter; *YNI1*, nitrite reductase; *YNA1*, Zn(II)_2_Cys_6_ transcriptional activator; *YNR1*, nitrate reductase; *YNA2*, Zn(II)_2_Cys_6_ transcriptional activator. **B. Northern blots showing the effect of different nitrogen sources on the expression of the nitrate assimilation genes in the *H*. *polymorpha* wild type strain (L1).** The actin gene mRNA signal was used as loading control. Nitrogen sources were 50mM alone or in combinations as indicated above the autoradiographs. N, nitrate; A, ammonium; Q, glutamine; E, glutamate; P, proline. Mean densitometric values (± standard error) of separate experiments, normalised to the loading control signal, are reported (expressed as percentage of maximum induction value i.e., growth on nitrate). nd, not-detectable. **C. Northern blot analysis investigating the role of *YNA1* and *YNA2* in the expression of the nitrate cluster genes.** Mutant strains are derived from the wild type (L1) and carry either a marker insertion at the *YNA1* locus (L1-yna1^I6632^) or maker insertion and internal deletion at the *YNA2* locus (L1-yna2^Δ12274–12595^). The actin gene signal was used as loading control.

The ascomycete yeast *Hansenula polymorpha* (syn. *Pichia angusta*) is able to use nitrate as sole nitrogen source [1]. The genes involved in nitrate assimilation are inducible by the substrate and encode nitrate reductase (*YNR1*), nitrite reductase (*YNI1*) and a high affinity nitrate transporter (*YNT1*). These co-regulated genes reside in a cluster together with the two pathway-specific regulatory genes *YNA1* and *YNA2* [2] (Fig 1A).

Nitrate transport has a key role for the entrance of the inducer in the cell where the transporter acts as a modulator that triggers nitrate gene expression. Unlike *Arabidopsis thaliana*, where the nitrate transporter CHL1 plays the role as nitrate sensor independently to that of nitrate transporter, *H*. *polymorpha* Ynt1 and *A*. *nidulans* NrtA/NrtB do not show this sensor activity [1,3,4]. As in *A*. *nidulans* and *N*. *crassa*, also in *H*. *polymorpha* the expression of the transporter and enzymes involved in nitrate assimilation is subject to both nitrate induction and nitrogen metabolite repression, which blocks the nitrate pathway when the products of the reduction process such as ammonium or glutamine, are available in the growth medium [2,5–13]. Depending on the nitrogen source, Ynt1 is subject to post-translational modifications by ubiquitination and phosphorylation. Recently, Martìn and co-workers (2011) identified and described the *NPR1* gene, encoding the Npr1 (Nitrogen Permease Reactivator 1) kinase involved in Ynt1 phosphorylation and regulation in response to the nitrogen and carbon source availability [14,15].

The *YNA1* and *YNA2* regulatory genes, also located in the nitrate utilization cluster, encode two binuclear Zn(II)_2_Cys_6_ *trans*-activators involved in transcriptional activation of genes necessary for nitrate utilization [5,6]. These factors belong to the same fungal-specific protein family as the *Aspergillus nidulans* NirA [16] and *Neurospora crassa* NIT4 [17] proteins even if they are not their orthologues [18]. The latter reference also discusses the variation in clustering in different fungi and the transcription factors associated with the nitrate assimilation cluster in different yeasts able to assimilate nitrate. Yna1p shows a uniquely deviant Zinc cluster motif with seven amino acids, instead of six, between the second and the third cysteine [19,20]. C-terminal of the DNA binding domain Yna2p contains a region of periodically spaced leucines which may form a leucine-zipper domain involved in protein-protein interactions. Interestingly, NirA contains a similar motif and there it was shown to be essential for *in vitro* binding to cognate DNA sequences [21].

In eukaryotes, the chromatin structure plays an essential role in the modulation of transcriptional regulation [22–26], where chromatin remodelling enables interaction between the transcriptional machinery and the target DNA sequences [24,27–29]. Among the Zn(II)_2_Cys_6_ transcription factors, Gal4p of *S*. *cerevisiae* directly promotes chromatin remodelling associated with expression of their target genes [29–32]. Chromatin transitions in nitrate assimilation pathways were also studied in *N*. *crassa* [33] and *A*. *nidulans* [34–37]. Interestingly, in *N*. *crassa* the formation of hypersensitive chromatin regions at the *nit-3* gene promoter (coding for nitrate reductase) is mediated by both regulators NIT4 (nitrate-specific transcription factor) and NIT2 (mediating nitrogen metabolite de-repression) independently of the induction process [31]. In *A*. *nidulans*, while both nitrate induction and AreA are required to restructure the chromatin landscape in the bidirectional *niiA-niaD* promoter [37], NirA (the NIT4 ortholog) is only required for sliding nucleosome -1 and + 1 around the central binding sites for both NirA and AreA. Therefore, the majority of chromatin reorganisation depends on AreA and can take place independently from transcriptional activation of the cognate genes. Some years ago we have shown that one function of AreA is to recruit histone acetylation activities to the bidirectional *niiA*-*niaD* promoters under nitrogen metabolite de-repressing conditions, i.e. in the absence of repressive nitrogen sources such as glutamine or ammonium [11,12,37].

In this study we identified differential roles for Yna1 and Yna2 in the transcriptional activation events. In particular, we gained insights into (i) their subcellular localization, (ii) *in vitro* and *in vivo* binding to putative target sites (iii) nucleosomal positioning dynamics at the *YNT1* promoter in response to different physiological conditions.

## Materials and Methods

### Yeast strains

All strains used in this work are derivatives of *H*. *polymorpha* homothallic haploid NCYC 495. The NCYC495 derivative strain L1 (*leu1-1)* [38] was used as recipient strain to obtain the *YNA1* and *YNA2* null mutants, respectively denoted L1-*yna1* ^*16632*^ and L1-*yna2* ^*Δ12274–12595*^. L1 was used as wild type strain throughout this work. Mutant strains L1-*yna1* ^*16632*^ *(leu1-1*, *yna1* ^*16632*^::*LEU2)* and L1-*yna2* ^*Δ12274–12595*^ (*leu1-1*, *yna2* ^*Δ12274–12595*^::*LEU2)* were constructed by standard methods. L1-*yna1* ^*16632*^ was obtained by target integration of a 4095 bp- DNA fragment containing the *Candida albicans LEU2* gene flanked by *YNA1*. The 4095 bp DNA fragment (LD-I cassette; S2 Fig) was amplified by PCR from the pYNA1LEU-I plasmid using primers CCYNA1F and CCYNA1R (S1 Table). Transformants containing the *yna1* ^*16632*^::*LEU2* mutation were selected as Leu^+^ Nit^-^ strains, and the presence of a single homologous integration was checked by PCR and Southern blots. L1-*yna2* ^*Δ12274–12595*^ was constructed by substitution of the 322 bp *Cla*I-*Cla*I fragment of the *YNA2* coding region, with the *Candida albicans LEU2* gene. The DNA fragment containing the *Candida albicans LEU2* gene flanked by *YNA2* (D2 cassette; S3 Fig) used to obtain this mutant by target integration was amplified by PCR from the pYNA2LEU plasmid using primers YNA2Ampf and YNA2AmpR (S1 Table). Transformants, containing the *yna2* ^*Δ12274–12595*^::*LEU2* mutation, were selected as Leu^+^ Nit^-^ strains and the presence of a single homologous integration was checked by PCR and Southern blots.

The *Escherichia coli* strains MC1061 *[hsdR mcrB araD139D (araABC-leu) 7679 dlacX74 galU galK rpsL thi]* and DH5α [fhuA2 Δ(argF-lacZ)U169 phoA glnV44 Φ80 Δ(lacZ)M15 gyrA96 recA1 relA1 endA1 thi-1 hsdR17] were used for routine plasmid preparation.

### Growth conditions

All *H*. *polymorpha* strains were grown at 37°C in rich medium (YPD) containing 2% glucose, 1% yeast extract and 2% peptone, or in a minimal medium (MM) containing 2% glucose, 0.2% yeast nitrogen base lacking of both amino acids and ammonium sulphate (Difco, Detroit, MI, USA), plus 0.5% ammonium sulphate (corresponding to 75mM NH_4_^+^) or 50 mM of proline, glutamine or glutamate. When necessary, 1.8% agar or 0.006% leucine were added. The standard growth conditions for northern blots and MNase digestion were as follows: cells were grown in 250 ml flasks with 50 ml medium in an orbital incubator at 37°C and 200 rpm. Pre-culturing in YPD for 15 hours, the cells were washed with sterile water and inoculated (10^6^ cells/ml) on minimal medium containing 50 mM proline as nitrogen source. After 16 hours culture the cells were again washed with sterile water and then transferred to a MM containing one of the following nitrogen sources: 50 mM NaNO_3_, 50 mM NH_4_Cl, 50 mM L-glutamine, 50 mM Na-glutamate, 50 mM L-proline, 50 mM NH_4_NO_3_, 50 mM NaNO_3_ + 50 mM L-glutamine, 50 mM NaNO_3_ + 50 mM Na-glutamate, 50 mM NaNO_3_ + 50 mM L-proline. After the optimal repressing conditions were established, four standard conditions were used: 50 mM L-proline (not-induced-de-repressed condition, *NI)*; 50 mM NaNO_3_ (induced-de-repressed conditions, *I)*; 50mM L-glutamine (not-induced-repressed condition, *R)*; 50 mM L-glutamine + 50 mM NaNO_3_ (not-induced-repressed conditions, *IR)*. When not otherwise stated, cultures were further incubated for 2 hours and then harvested.

The growth conditions for *in vivo* footprinting of *H*. *polymorpha* were as follows: cells were grown in 500 ml flasks with 100 ml of MM containing 50 mM proline as nitrogen source at 37°C and 180 rpm. After pre-culturing for 15 hours, cells were washed with sterile water and inoculated (106 cells/ml) in MM containing one of the following nitrogen sources: 50 mM L-proline (not-induced-de-repressed condition, *NI)* or 50 mM NaNO_3_ (induced-de-repressed condition, *I)*. Cultures were further incubated for 2 hours. Guanines and adenines methylation of genomic DNA in whole and living cells was performed by adding to each flask 10 ml of a solution containing 9,925 ml of 20 mM MES buffer pH 6.2 and 75 μl of pure dimethylsulphate (DMS, Sigma-Aldrich). Cells were incubated for 2 minutes at 37°C and 180 rpm. Methylation was stopped by adding TLE-β stop buffer (Tris-Cl pH 8.0, 10 mM; EDTA pH 8.0, 1 mM; LiCl 100 mM; β-mercaptoethanol 2%). Cells were then harvested for genomic DNA extraction.

Growth conditions for *H*. *polymorpha* strains L1-GFP-1 and L1-GFP-M2 harbouring the chimeric constructs *YNA1(p)*::*YNA1*::GFP and *MOX(p)*::*YNA2*::GFP respectively, were as follows: cells were pre-cultivated in 100 ml flasks with 20 ml of MM supplemented by 50 mM proline as nitrogen source, at 37°C and 180 rpm. Cells were washed with sterile water and resuspended in 20 ml of MM w/o nitrogen source. 3 ml of each sample was aliquotted in a 15 ml-tube containing MM plus 50 mM of nitrogen source related to the physiological condition under investigation. Cells were then incubated at 37°C, 180 rpm, at different times (from 20 minutes to 2 hours) and observed by confocal microscopy.

The *H*. *polymorpha* strain AL-YFP18, harbouring the chimeric cassettes *YNA1(p)*::*YNA1*::YFP^B^::*LEU2*::*YNA1(t)* and *YNA2(p)*::*YNA2*::YFP^C^::*PUR7*::*YNA2(t)*, was pre-grown in 100 ml flasks containing 20 ml of MM supplemented by 50 mM Glutamine as sole nitrogen source. Cells were washed with sterile water and resuspended in 20 ml of MM w/o nitrogen source. 3 ml of each sample was aliquotted in 15 ml-tube containing MM plus 50 mM of nitrogen source related to the physiological condition under investigation. Cells were incubated at 37°C, 180 rpm for 2 hours and observed by confocal microscopy.

For chromatin immunoprecipitation (ChIP) assays with the GFP antibody, strains L1-*yna1* ^*16632*^ and L1-*yna2* ^*Δ12274–12595*^ harbouring the *YNA1(p)*::*YNA1*::GFP and *MOX(p)*::*YNA2*::GFP constructs, respectively, were grown at 37°C, 180 rpm, on MM containing ammonium as nitrogen source and glucose as carbon source. After 18 h, cells were harvested, washed and cultivated for additional 2 hours on MM containing nitrate (I) or glutamine (R). For strains harbouring the construct *MOX(p)*::*YNA2*::GFP, methanol was used as carbon source and inducer.

### Plasmids

Plasmid pA1 contains *YNA1* amplified using primers YNA1ef and YNA1dr and inserted in *Eco*RI of EBOX37p6. pA2 contains the *YNA2* gene, amplified using primer YNA2df and YNA2dr cloned in *Bam*HI of EBOX42p1. EBOX37p6 and the EBOX42p1 are two *H*. *polymorpha* replicative plasmids [*PMR1* (GeneBank # AJ011906), *H*. *polymorpha LEU2*, *E*. *coli* ori; *KanR* (EBOX37p6) or *AmpR* (EBOX42p1)]. The pYNA1LEU-I plasmid, containing the LD-I cassette (S2 Fig), derives from pTZ18 (Pharmacia) and was constructed as follows: a 2352 bp DNA fragment containing the *YNA1 locus* and its regulatory regions was amplified using the YNA1df and YNA1dr primers, that contain the *Sal*I and the *Eco*RI restriction sites, respectively, at their 5’ ends. Genomic DNA was used as template. The amplified fragment was cloned into pTZ18 previously cut with *Sal*I and *Eco*RI. Then, a 2122 bp DNA fragment containing the *Candida albicans LEU2* was amplified from the pMK155 plasmid (a gift from R. D. Cannot; University of Otago, New Zealand) using primers leu2cf and leu2cr and cloned into the *Eco*RV restriction site of this intermediate construct (*Eco*RV is in the *YNA1* gene), the latter having been previously linearized with *Eco*RV and then dephosphorylated. The *LEU2* containing PCR fragment was cut with *Sma*I and then ligated with the plasmid. The construction was confirmed by restriction analyses and sequencing (automatic sequencing; MWG biotech, Ebersberg, Germany). The pYNA2LEU plasmid, containing the D2 cassette (S3 Fig), derives from pTZ18 (Pharmacia) and was constructed as follows: a 2777 bp DNA fragment containing *YNA2* was amplified using the primers YNA2df and YNA2er, which contain *Bam*HI at the 5’ ends. Genomic DNA was used as template. The amplified fragment was cloned into *Bam*HI linearized pTZ18. Successively, this intermediate construct was cut with *Cla*I; the *Cla*I sticky ends were filled-in using the Klenow DNA polymerase I fragment (Promega, Madison, WI, USA), dephosphorylated and finally ligated with a *Sma*I/*Sma*I-end 2122 bp DNA fragment containing the *Candida albicans LEU2* gene obtained as described above. The construction was confirmed by restriction analyses and sequencing.

For the expression of Yna1p_(1–185)_ and Yna2p_(1–180)_ truncated forms in *E*. *coli*, *YNA1*_*(1–555)*_ and *YNA2*_*(1–540)*_ were cloned by Gateway Cloning Technology, following manufacturer instructions. Yna1p_(1–185)_ and Yna2p_(1–180)_ contain putative nuclear localisation signals, activation domains and Zn(II)_2_Cys_6_ binding domains. In addition, Yna1p_(1–185)_ harbours a putative nuclear export signal (NES), which is not predicted for Yna2p. *YNA1*_*(1–555)*_ and *YNA2*_*(1–540)*_ were amplified from plasmids by using primers GAT*YNA1*_fw, GAT*YNA1tr*_rv and GAT*YNA2_*fw, GAT*YNA2*tr_rv respectively. pDEST17 destination vector was used as final expression vector in *E*. *coli* BL21DE3 strain. Gene expression is driven by *E*. *coli* T7 promoter and mediated by T7 RNA polymerase.

Plasmid pH1 (Lab. E. Berardi, auto-replicative vector Kanr, *ScLEU2*, *MOX(p)*::eGFP-Tamo derived from pHIPX4, [39] was used for *YNA1(p)*::*YNA1*::GFP and *MOX(p)*::*YNA2*::GFP fusions. A *Sma*I/*Not*I *YNA1*-fragment of 2219 bp and a *Bam*HI/*Sma*I *YNA2*-fragment of 2206 bp were amplified by PCR using genomic DNA as template. The chimeric fragment *YNA2*::GFP was assembled under the control of methanol-inducible promoter *(MOX)* from *H*. *polymorpha* methanol-oxidase gene because of the weak fluorescent signal detectable under control of *YNA2* native promoter. Primers YNA1gfp_fw, YNA1gfp_rv, BamYNA2gfp_fw and YNA2dr were used. *YNA1* and *YNA2* PCR products were respectively cloned into *Sma*I/*Not*I and *Bam*HI/*Not*I restriction sites in the linearized plasmid pH1. Constructions were confirmed by restriction analysis, PCR amplification and sequencing (Agowa DNA sequencing, GmbH). All primers used are indicated in S1 Table.

### Bimolecular fluorescence complementation

Bimolecular fluorescence complementation is a technology used to prove the interaction of two proteins belonging to the same macromolecular complex. In this work, Yna1p and Yna2p have been fused to unfolded complementary fragments of yellow fluorescent protein (YFP) and expressed in the *H*. *polymorpha* control strain. Firstly, a cassette for C-terminal genomic integration for both *YNA1* and *YNA2* was constructed by fusion PCR. Each cassette harbors the ORF C-terminal and the terminator sequences of the target gene as flanking regions, the YFP unfolded fragment (YFP^B^ for *YNA1* and YFP^C^ for *YNA2*) and the selection marker. As selection markers, *CaLEU2* and *PUR7* were used. *YNA1* and *YNA2* cassettes were expressed in L1 (*leu1*.*1*, *mat a*) and in A11 (*ade11*.*1*, *mat α*) strains, respectively. L1 and A11 were crossed and sporulated on ME plates as described [40]. Spores were analyzed by PCR and phenotype selection. The expression of each construct integrated in the *YNA* locus is under the control of the native promoter. The resulting strain AL-YFP18 carrying *YNA1(p)*::*YNA1*::YFP^B^::*CaLEU2*::*YNA1(t)* and *YNA2(p)*::*YNA2*::YFP^C^::*PUR7*::*YNA2(t)* genomic modifications was pre-cultivated for 16–18h in MM containing glutamine as sole nitrogen source. After pre-cultivation, cells were harvested, washed three times and cultivated in minimal medium containing nitrate (induction, I) or proline (not-induction-de-repression, NI) or glutamine (repression) as nitrogen source. After 2h, cells were observed by confocal microscopy.

### Molecular techniques

Standard recombinant DNA techniques were carried out according to Sambrook *et al*. [41]. Transformation of *H*. *polymorpha* was performed by the lithium acetate method described by Berardi and Thomas [42]. Restriction enzymes and biochemicals were obtained from Promega (Madison, WI, USA), Fermentas (Burlington, Ontario, Canada) and Sigma-Aldrich (St. Louis, Missouri, USA) and used as detailed by the manufacturer.

### Southern blot analysis

Southern blot analyses were carried out as in Sambrook *et al*. [41]. The probes, labelled with ^32α^dCTP using a Random Priming Kit (Amersham Pharmacia Biotech, Piscataway, NJ, USA), were obtained by PCR using the following primers: probe-*Y1*, YNA1SF (forward) and YNA1SR (reverse); probe-*Y2*, sout2F and sout2R (S1 Table).

### Northern blots

Total RNAs were isolated from cells as described by Schmitt *et al*. [43]. Equal amounts (10 μg) of total RNA were loaded per lane and separated on glyoxal agarose gel. Blots were hybridised with the *YNT1*, *YNI1*, *YNR1*, *YNA1* or *YNA2* probes labelled with ^32α^dCTP using a Random Priming Kit (Amersham Pharmacia Biotech, Piscataway, NJ, USA). The actin probe (*HpACT)* [44] was used to normalize the amounts of RNA loaded on a blot. All probes used to detect the different transcripts were amplified from genomic DNA by PCR using the following primers: *YNT1*, YNT1SF and YNT1SR; *YNI1*, YN2SF and YN1SR; *YNR1*, YNRSF and YNR1SR; *YNA1*, YNA1SF and YNA1SR; *YNA2*, YNA2ST and YNA2SF; *HpACT*, ACTesa (forward) and ACT1HPF (reverse) (S1 Table). Northern blots were quantified using a PhosphorImager with ImageQuant software (Molecular Dynamics, Sunnyvale, CA, USA). Northern blots presented are typical examples from at least two experiments. When quantitative measures are shown these are averages and standard errors from 5 independent determinations.

### Low resolution micrococcal nuclease mapping

Micrococcal nuclease (MNase) sensitivity analyses were carried out by the indirect end labelling technique, using the method described for filamentous fungi by Gonzalez and Scazzocchio [36]. This method was adapted to yeast *H*. *polymorpha* as indicated below. The cells were grown as indicated above and harvested by centrifugation. The remaining water was eliminated by a rapid filtering through a sterile membrane filters (1,2 μm; Millipore, Bedford, MA, USA). Successively the cell-cream was immediately frozen in liquid nitrogen and then reduced to powder by grinding under liquid nitrogen in a mortar. The powder was suspended in MNase digestion buffer to a final concentration of 100 mg of cell powder/ml (nystatin was added to a final concentration of 50 μg/ml). MNase digestion was started by the addition of different amounts of the enzyme to 200 μl of this suspension, and then incubated for 10 min at 37°C. The reaction was stopped by adding 1 vol. of 40 mM EDTA, 2% SDS. The mixture was extracted with phenol-chloroform, then treated with 10 mg/ml RNase A (10 min.– 37°C), precipitated from 2 vol of ethanol and the pellet suspended in 50 μl of TE pH 7.4.

In preliminary experiments the nucleosomal repeat length was estimated at 145 bp, consistent with that previously reported by Costanzo and coworkers [45] for *H*. *polymorpha*. The MNase concentrations varied in a range 3.75–7.5 U/g of cell powder. Naked DNA samples were digested with MNase (from 0.003 to 0.05 U/μg of DNA) in 200 μl of digestion buffer (7 min, 30°C) and then processed as described for the other samples above.

For low resolution mapping of nucleosomes by indirect end labelling the purified DNAs were subject to a secondary digestion by *Eco*RV. The specific DNA sequences were detected by hybridisation with the *YNI1* probe that is the 348 bp *Eco*RI-*Eco*RV fragment from the I*-1* PCR product. The probe is labelled with ^32α^dCTP using a Random Priming Kit (Amersham Pharmacia Biotech, Piscataway, NJ, USA). The I*-1* PCR product was obtained using the primer forward croYNIF and croYNIR (S1 Table). Blotting and hybridization were as in Wu (1980) [46]. For each strain and growth condition experiments were performed at least in duplicate. In order to determine the size of each DNA band and to make an accurate comparison of samples run on different gels, a Pharmacia 100 bp ladder and a Smart Ladder (0.2 Kbp to 10 Kbp; Eurogentec, Seraing, Belgium) were included in every gel. In all cases, gels were scanned and the size of the bands estimated using the Molecular Analyst/Macintosh Software (Bio-Rad, Hercules, CA, USA), with the ladders as standards.

### *HIS*-Yna1p_(1–185)_ and *HIS*-Yna2p_(1–180)_ purification

*HIS*-Yna truncated proteins expressed in *E*. *coli* were purified by nickel affinity chromatography (NTA-Ni ProBond resin), as indicated by manufacturer. Protein elution from ProBond resin was done by using an imidazole gradient from 10 to 200 mM. Protein concentration was determined by colorimetric Bradford assay (λ = 595 nm) and spectrophotometric evaluation (λ = 280 nm), using BSA standard curve as reference.

### Band shift assay

75 ng of purified proteins were incubated on ice in 10 μl of KCl 1mM and Spermidine 80 mM in presence of both non-specific [0,1 mg/ml poly(dIdC)] and specific competitors. [^32^P]ATP-end-labeled YNT1 oligonucleotide (YNT1-oligo, S1 Table) was added and the mixture was incubated 30 min at room temperature. DNA-protein complexes were separated for 2–3 h in 6% polyacrylamide gel (30% AA/0,8% b-AA) containing 50% glycerol and 0,5x TBE. After drying in a vacuum heater (Gel dryer, Model 583, Bio-Rad), the gel was exposed overnight on intensifying screens (Storage Phosphor Screen, Molecular Dynamics).

### *In vivo* footprinting

Growth conditions for *H*. *polymorpha* were indicated above. *In vivo* footprinting method for *Aspergillus nidulans* [47] was adapted to *H*. *polymorpha*. After extraction of methylated DNA, a 10-fold diluted piperidine (Roth, Germany) was added to each DNA aliquot. Samples were incubated for 3 minutes at 90°C and cooled on ice. *In vitro* methylation was performed by dissolving DNA in 200 μl of MES buffer and 1 μl of DMS and incubating the mixture at 90°C for 90 s. After ethanol precipitations, DNA was resuspended in TE at final concentration of 0.7 mg/ml. A sample DNA aliquot was loaded on agarose gel to confirm the existence of a smear containing fragments in a range of 0–1000 bp. Methylated and piperidine treated DNA samples were analysed by LMPCR [47], using Vent polymerase (NEB). [^32^P]ATP-labelled amplification products were separated on a sequencing gel which was dried and exposed to intensifying screens. To investigate the *H*. *polymorpha YNT1* promoter, Primer1_sense, Primer2_sense, Primer3_sense were used (S1 Table).

### Chromatin Immunoprecipitation (ChIP)

*H*. *polymorpha* cells were grown as indicated above and treated with formaldehyde at a final concentration of 1% for 20 min at 37°C and shaking at 180 rpm, and subsequently, glycine was added to a final concentration of 2,5 M and incubated for additional 10 min. Cells were harvested and washed with ice-cold PBS and subsequently treated for 30 min at 30°C with a buffer containing Zymolyase to obtain spheroplasts. Cells were harvested by centrifugation and resuspended in lysis buffer. Each sample was sonicated at 20–22 amplitude for 5 times, 5 min each, with intervals of 1 min. Cell extracts were immunoprecipitated overnight using magnetic beads (Dynabeads Protein G and Dynabeads Protein A, Novex, Life Technologies) coated with Ab290 Abcam anti-GFP antibody. In the next step, each mixture was washed and the Yna::GFP-DNA complexes eluted using TES buffer at 65°C. Samples were treated with Proteinase K for 1,5 h and DNA isolated by phenol-chloroform extraction and 2-propanol precipitation. Each pellet was eluted in 30 μl of Tris-EDTA solution. Samples were analysed by quantitative PCR, using different concentrations of genomic DNA ranging from 10 ng to 10 pg as serial dilutions to generate a standard curve. The sequence of the primers YT_Ch_fw and YT_Ch_rv used to amplify a region of 114 bp containing the Yna1/Yna2 binding site are indicated in S1 Table. The PCR cycle used is as follows: 95°C, 3 min; 50x(95°C, 30 sec; 60°C, 20 sec; 72°C, 30 sec); 95°C, 15 sec. Relative amounts of precipitated DNA are expressed as ratio between the amount of DNA obtained after ChIP relative to the amount of DNA contained in the respective sample before precipitation (input DNA).

### Confocal microscopy

Growth conditions for *H*. *polymorpha* cells were as indicated above. 5 μl of each culture were spotted on a cover slide. For Yna1p::GFP and Yna2p::GFP subcellular fluorescence detection, a Leica TCS SP2 confocal microscope was used (GFP excitation 488 nm, Ar-Kr laser; GFP emission measured between 500 and 555 nm). For YFP (excitation 514 nm, emission 527 nm) and DAPI (excitation 350 nm, emission 470 nm) detection an Olympus FV1000 microscope was used.

## Results

### Regulation of the nitrate utilisation cluster

Similarly to other fungi, transcriptional activation of nitrate assimilation genes in *H*. *polymorpha* requires the presence of nitrate and, at the same time, the absence or low levels of reduced nitrogen sources [5,6,48–50]. To set the appropriate experimental conditions for subsequent molecular analyses, we first incubated cells under a variety of nitrogen conditions. We also investigated whether different reduced nitrogen compounds afford different levels of nitrogen metabolite repression when present at equimolar amounts in the medium simultaneously with the inducer. Fig 1B shows that glutamine is the most efficient repressing source, followed by glutamate and ammonium. All the nitrate cluster genes, with the exception of the constitutively transcribed *YNA2* gene are strongly repressed by glutamine, whereas addition of ammonium does not result in complete repression. The least repressive effect is exerted by proline as the steady state mRNA levels on proline plus nitrate are from 40% to 85% of those obtained upon incubation on nitrate alone. This indicates that nitrogen catabolite repression (NCR) depends on the metabolic rate by which a given metabolite is converted to the possibly direct effector for NCR, glutamine. Induction of the structural nitrate utilisation genes (*YNT1*, *YNR1*, *YNI1*) has a considerable lag-time of around 40 minutes whereas the regulatory gene *YNA1* is already transcribed after 20 minutes of transfer to nitrate (S1 Fig). *YNA2* levels show an expression peak 20 minutes after the transfer to nitrate (S1 Fig). Our subsequent work was carried out (unless otherwise indicated) under four physiological conditions, (i) not-induced-de-repressed, NI (proline); (ii) induced-de-repressed, I (nitrate); (iii) induced-repressed, IR (nitrate + glutamine); (iv) not-induced-repressed, R (glutamine).

### *YNA1* and *YNA2* form a positive feedback loop

As expected from previous work, both *YNA1* and *YNA2* mutants (L1-*yna1* ^*I6632*^ and L1-*yna2* ^*Δ12274–12595*^) were unable to utilise nitrate or nitrite as nitrogen sources due to the lack of expression of assimilatory and transporter genes (Fig 1B and 1C). Each mutation was complemented by a replicative plasmid carrying respectively the *YNA1* or the *YNA2* gene (see Materials and Methods and S2 and S3 Figs). As shown in Fig 1C, a null mutation in either *YNA1* or *YNA2* results in non-detectable expression of *YNR1*, *YNI1* and *YNT1* under all growth conditions tested. The only exception was *YNT1*, which shows very low, albeit detectable expression on nitrate in the *YNA2* mutant. In their initial characterization of *YNA1* and *YNA2* Ávila and co-workers [6,51] found that *YNA1* expression is independent from *YNA2*. Yet in our slightly different genetic background (NCYC495^–leu^) this is only true for nitrate as sole nitrogen source, when other nitrogen sources were employed (e.g. proline plus nitrate, ammonium plus nitrate), the absence of *YNA2* led to a defect in *YNA1* expression. This clearly indicates that Yna2p is required for full expression of its partner activator in this genetic background.

### Yna1p nuclear localization is Yna2p-dependent

**Fig 2:**
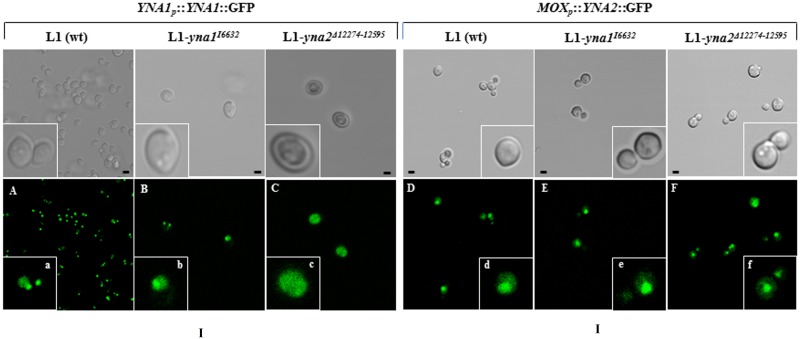
Nuclear accumulation of Yna1p is Yna2p-dependent. **A, B, C.** Yna1p expressed as a GFP fusion protein from its own promoter in the L1 wild type strain and the **L1-*yna1*** ^***I6632***^ **and L1-*yna2*** ^***Δ12274–12595***^ **null mutants is shown. Pictures of this figure were taken from strains incubated on nitrate but identical results were obtained on all media, i.e. also on non-inducing (proline), inducing-repressing (nitrate and glutamine) or repressing (glutamine) conditions**. Photographs of the cultures incubated on proline, nitrate and glutamine or only glutamine are shown in S4 Fig. In the inlet of each photograph a higher magnification shows details of one representative cell of the experiment. **D, E, F.** Yna2p-GFP localization in a strain expressing the fusion gene from the MOX promoter (to be able to detect the protein also in a *YNA1* mutant). A detail for each strain is shown as inlet in the photographs. Scale bars refer to 2 μm.

To study whether the transcription factors are regulated by nuclear-cytoplasmic shuttling, the *YNA1* and *YNA2* genes were fused to GFP. *YNA1*-GFP expression was driven by its native promoter, whereas *YNA2*-GFP was under the control of the methanol-inducible promoter (*MOX(p)* from methanol oxidase gene). Heterologous *YNA2-*GFP expression by the *MOX* promoter was chosen because Yna2p-GFP should also be studied in *YNA1Δ* backgrounds and, as determined above, the *YNA2* gene is almost not expressed in a strain lacking Yna1p (L1-*yna1* ^*I6632*^, Fig 1C). Both constructs contained in an autonomous, self-replicating plasmid were transformed into the wild type, *YNA1Δ* and *YNA2Δ* backgrounds and strains were grown under all relevant physiological conditions. To detect Yna2p::GFP, the fusion gene was always expressed to high levels using methanol as inducer. Confocal microscopy of the transformants containing the GFP fusion constructs in the wild type background (labelled L1) revealed that both Yna1p::GFP and Yna2p::GFP are located predominantly in the nucleus under all physiological conditions tested, namely **non-inducing (NI, proline), inducing (I, nitrate), inducing-repressing (IR, nitrate and glutamine) or repressing (R, glutamine) conditions**. Fig 2, panels A-F, show confocal microscopy images and magnifications of a representative cell of the image as inlets (a-f) of the wild type and mutant strains incubated on nitrate. Images from other conditions are shown in the supplementary information (S4 Fig). The fact that Yna1p::GFP is detected at all on glutamine is remarkable as *YNA1* mRNA is barely detectable under these repressing conditions (Fig 1B, “Q” conditions). This indicates that the fusion protein must be very stable. In the *YNA1Δ* strain (L1-*yna1* ^*I6632*^), Yna2p::GFP also locates constitutively and predominantly to the nucleus (Fig 2E), indicating that Yna1p does not play a significant role to bring Yna2p to the nucleus or to keep it there.

This demonstrates that the predicted putative NLS in Yna2p is functional under all physiological conditions, either in the presence or the absence of the co-activator Yna1p. This is in contrast to the dynamics of Yna1p subcellular distribution: in the *YNA2Δ* strain (L1-*yna2* ^Δ12274–12595^), Yna1p::GFP has lost its capacity to accumulate in the nucleus and uniformly distributes throughout the cell under all tested physiological conditions (Fig 2C and S4 Fig). This suggests that one function of Yna2p is to either facilitate import and/or to prevent export of Yna1p. This finding may provide an explanation for the *YNA2Δ* phenotype as in this strain the amount of accumulated Yna1p in the nucleus and thus transcriptional activity seems to be reduced.

### Yna1p and Yna2p physically interact on non-inducing and inducing, but not on repressing nitrogen sources

**Fig 3:**
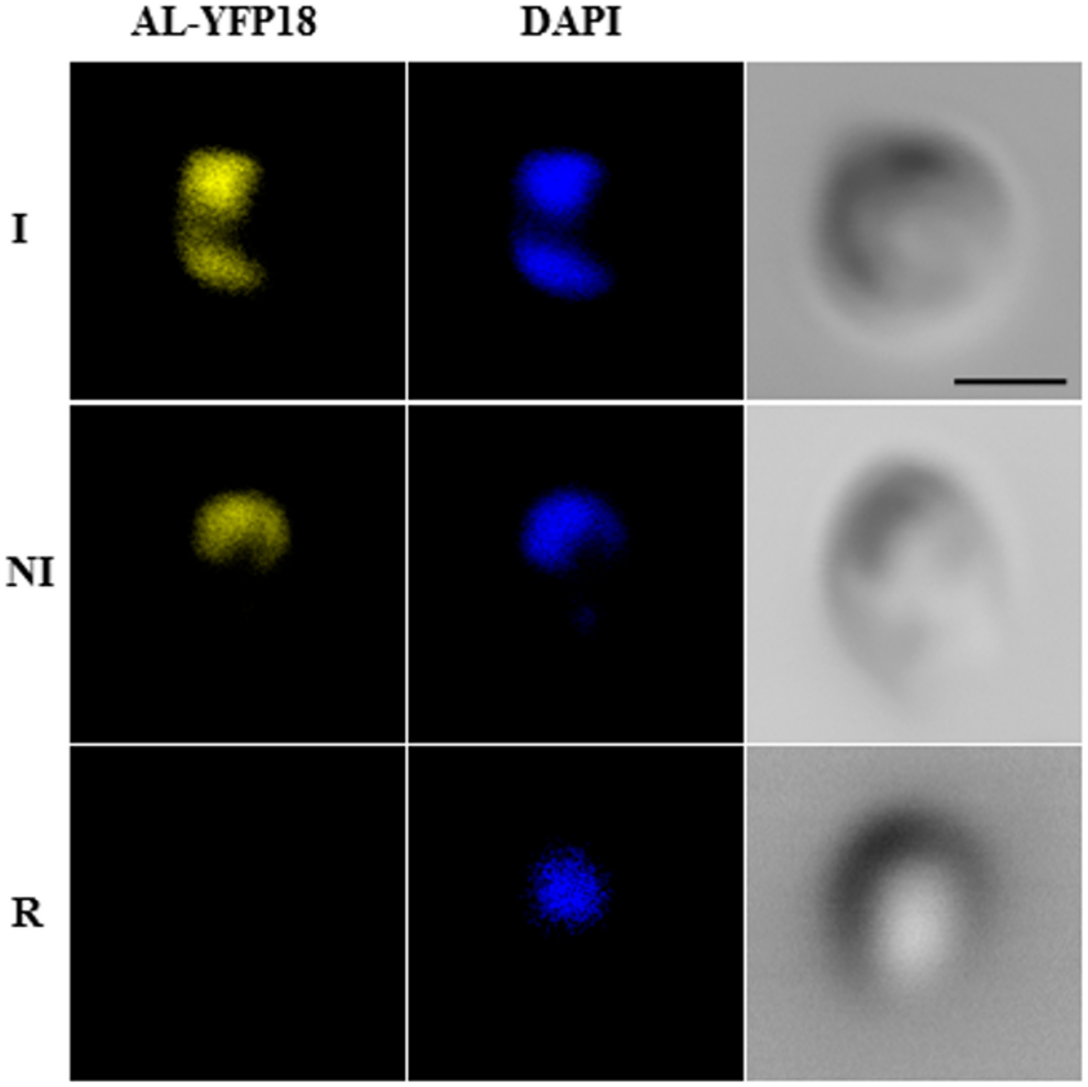
Yna1p and Yna2p interact *in vivo*. Fluorescence of the YFP protein is only visible in the reconstituted protein in the AL-YFP18 strain (wild type background) when both proteins Yna1p-YFP^Nterm^ and Yna2p-YFP^Cterm^ are simultaneously expressed (under nitrate-inducing, I, and proline non-inducing, NI, conditions) but is not detected under repressing conditions on glutamine (R). DAPI-staining detects the nuclei. Scale bars refer to 2 μm.

Results presented above suggest a direct interaction between Yna1p and Yna2p and such a complex may act as hetero-dimer to bind *cis*-acting elements to activate their target genes. To directly test this hypothesis we constructed split-YFP fusions of Yna1p::YFP^Nterm^ and Yna2p::YFP^Cterm^. The proteins were fused to partial fragments of YFP and in this case both fusion genes were transcribed from their native promoters (see Materials and Methods for details) and tested for their interaction. The appearance of yellow fluorescence at both non-induced and induced conditions indicates that the two proteins physically interact *in vivo* (Fig 3) and that this interaction is independent from the presence of the inducer.

This corroborates our previous results that nuclear accumulation of Yna1p depends on the presence of Yna2p which putatively regulates the function of Yna1p subcellular localization signals. In this context, Yna2p may either mask the Yna1p NES or be involved in exposing the NLS of Yna1p. A clear signal was only detected under inducing (I) and non-inducing conditions (NI) but was absent under repressing conditions (R, glutamine). This result could be due to the small amount of Yna1p::YFP, that is expected to be present in cells grown on glutamine although the Yna1p::GFP signal was readily detected on this N-source (compare S4 Fig). Alternatively, glutamine as N-source may disrupt the Yna1p-Yna2p interaction. The fact that Yna1p::GFP is readily detected might also indicate that the GFP signal requires less protein than detection of the YFP signal. Taken together, our studies on the subcellular localization and the split-YFP experiments suggest that physical interaction *between* Yna1p-Yna2p occurs *in vivo* and that this heterodimeric complex triggers transcriptional up-regulation of one of the regulatory genes (*YNA1*) as well as of the structural genes necessary for nitrate assimilation.

### Yna1p and Yna2p bind *cis*-acting sites in the *YNT1* promoter

**Fig 4:**
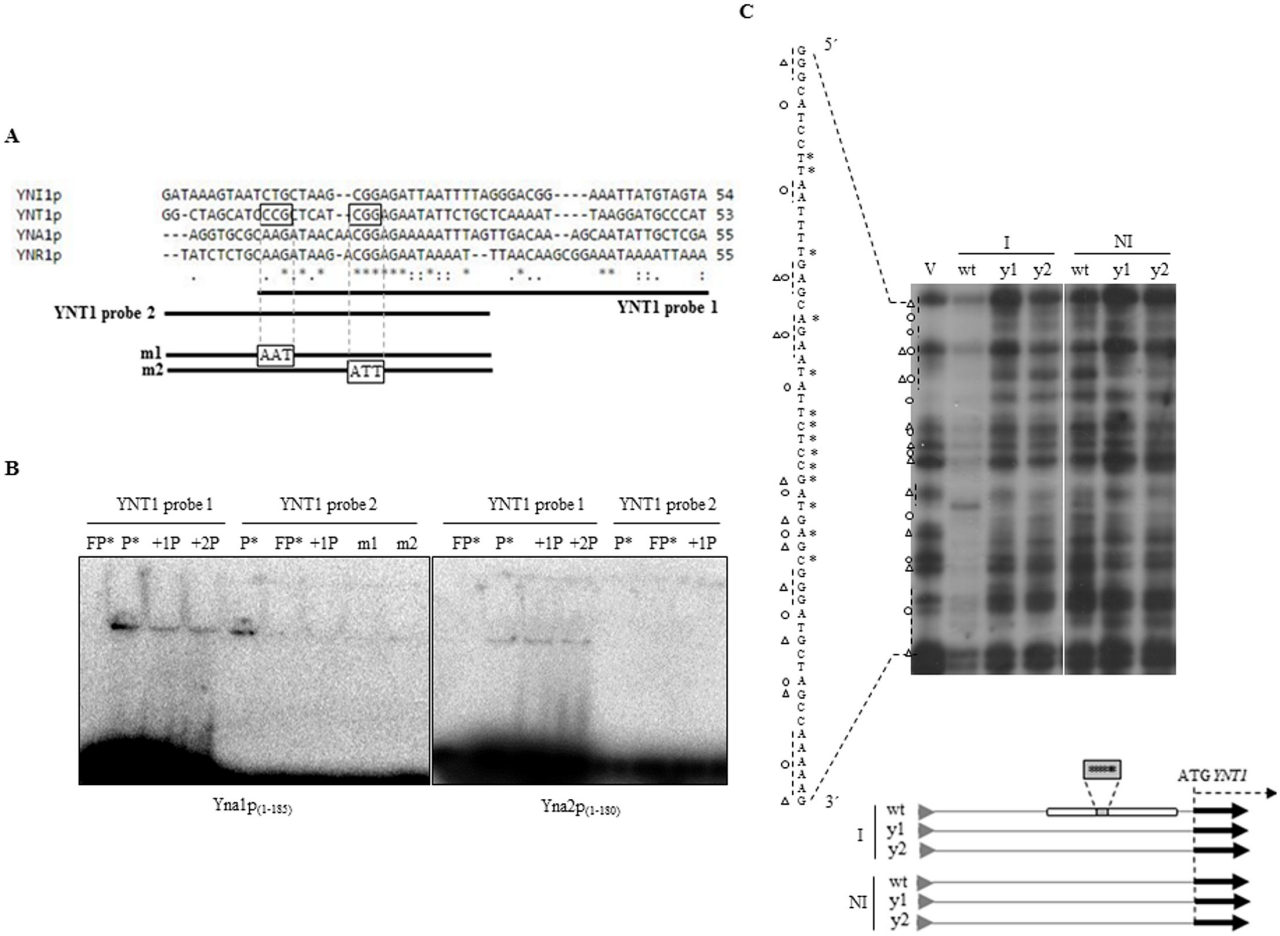
Yna1p and Yna2p bind *in vitro* and *in vivo* to a region containing a conserved DNA motif present in all nitrate-responsive promoters. **A.** Alignment of the promoter regions of genes belonging to the nitrate cluster detects a conserved motif (5′CGGAGA) indicated by six consecutive asterisks. This motif could represent a putative upstream activating sequence (UAS) bound by Yna1p and Ynap2. Probes used in the band shift assays are indicated as YNT1 probe 1 and YNT1 probe 2; the two different derivatives of YNT1 probe 2 containing the indicated changes in conserved nucleotides are shown as m1 and m2. **B.** *In vitro* interaction experiments using electric mobility shift assays (EMSA). FP*, labelled probe (double-stranded oligonucleotide as indicated in A) was incubated without recombinant proteins; P*, labelled probe incubated with the indicated recombinant protein but no unlabelled, specific competitor DNA has been added.; +1P, same conditions as in P* but an equimolar amount of the unlabelled double-stranded oligonucleotide was present in the incubation mixture as specific competitor for complex formation; +2P, same conditions as in +1P but double molar amount of the unlabelled double-stranded oligonucleotide was present in the incubation mixture as specific competitor. Radiolabelled probes described in panel A were incubated with recombinant *HIS*-Yna1p_(1–185)_ and *HIS*-Yna2p_(1–180)_ proteins expressed in *E*. *coli* and purified (as shown in S6 Fig). **While Yna1p**_**(1–185)**_ **binds both** *YNT1* probes 1 and 2, Yna2p_(1–180)_ doesn’t interact with the *YNT1* probe 2. Exchanging 3 nucleotides in the conserved 5′-CGGAGA-3′strech of YNT1 probe 2 disrupts the **Yna1p**_**(1–185)**_ **binding capability (m2). Likewise, exchange in an upstream conserved residue potentially forming a partial inverted repeat with the CGGAGA motif also disrupts complex formation (m1). This indicates that both motifs are involved in binding Yna1p. C.** *In vivo footprinting* methylation protection profile of the *YNT1* gene promoter. Cells from the wild type strain (wt) as well as from the *yna* mutant strains (y1, corresponding to **L1-*yna1*** ^***I6632***^ **, and y2, corresponding to L1-*yna2*** ^***Δ12274–12595***^ **) have been incubated at the standard physiological conditions** *(I*, *nitrate; NI*, *proline*) and subjected to ligation-mediated methylation protection footprinting as detailed in Materials and Methods. The sequence pattern of a control reaction containing *in vitro* methylated DNA is shown in lane V and the corresponding sequence derived from the *YNT1* promoter is aligned to the left of the autoradiograph. The sequence corresponding to the banding pattern in the autoradiograph lies between the diagonal dotted lines. In some cases guanines are not properly resolved in the gel but appear as compressed regions. Adenines can become hypersensitive and eventually appear as strong as guanines, mainly in the *in vivo* lanes. For a better orientation, vertical dotted lines next to the V lane (*in vitro* control reaction) and next to the sequence correspond to each other. Protected guanines are marked with a triangle and hypersensitive adenines are marked with circles. Asterisks on the displayed vertical sequence correspond to the asterisks marking the conserved residues in the *YNT1* promoter region used for designing *YNT1* probes 1 and 2. Note that the sequence displayed next to the autoradiograph is complementary to the sequence in panel A. Below the figure, the scheme shows the location of protected regions (white rectangles) in the *YNT1* promoter. The conserved 5′-CGGAGA-3′ sequence is indicated by the grey rectangle and asterisks.

An *in silico* analysis of the structural and regulatory gene promoters (Fig 4A) revealed the presence of a conserved, asymmetric motif 5′CGGAGA 3′ flanked by 3′AT-rich sequences in promoters of the inducible genes *YNT1*, *YNR1* and *YNI1*, as well as in the promoter of *YNA1*, but not in the constitutively transcribed *YNA2* gene.

Thus, we suspected that these sequences may mediate nitrate induction by providing recognition sites for the Yna1p and Yna2p proteins. On the basis of these nucleotide alignments, we first performed band shift experiments with recombinant forms of Yna1 and Yna2. We synthesized double-stranded, partially overlapping oligonucleotides representing the region -180 to -235 of the *YNT1* promoter as probes for the binding assays (Fig 4A). Both probes are similar in length (~ 40 nucleotides) and contain the conserved 5′CGGAGA 3′ motif, while probe 1 extends further towards the translation start point (-180 to -224), and probe 2 (-200 to -235) extends more distally into the promoter region. The truncated forms Yna1p_(1–180)_ and Yna2p_(1–185)_ containing the predicted Zn(II)_2_Cys_6_ DNA-binding domain, a putative dimerization domain and the predicted NLS (Nuclear Localization Signal; S5 Fig) were expressed as HIS-fusion proteins in *E*. *coli* and purified (S6 Fig). The purified proteins were subsequently used in the band shift experiments together with the radiolabelled probes. Both fusion proteins *HIS*-Yna1p and *HIS*-Yna2p bound to probe 1 containing the conserved motif and extending towards the transcription start, but t *HIS*-Yna2 did not bind probe 2 which lacks this latter region (Fig 4B). The formation of the DNA-protein complexes with both probes is specific for *HIS*-Yna1p under our conditions because an equimolar or two-fold excess of non-labelled probe significantly diminished (probe 1) or even abrogated (probe 2) the formation of the complex. This indicates that the *HIS*-Yna1p: probe 2 complexes are less stable than complexes formed with probe 1 containing a longer AT-rich stretch in the sequence towards the *YNT1* ORF. For *HIS*-Yna2p the scenario was even clearer, as this protein does not form a detectable complex with probe 2 lacking the 3′AT-rich sequences. These data indicate that *in vitro* both proteins are able to independently form stable and specific complexes with DNA when both the conserved 5′CGGAGA3′ motif as well as the 3′AT-rich sequences are available.

### *In vivo* footprinting reveals an extended region protected by Yna1p and Yna2p

We then determined which sequences in the *YNT1* promoter are targeted *in vivo* and under which conditions putative recognition sequences interact with the activators. To this end, we performed *in vivo* methylation protection footprinting experiments to single out the purines protected by a DNA-binding protein under a given physiological condition [47]. Again, we targeted the *YNT1* promoter. The pattern obtained after ligation-mediated PCR is shown in Fig 4C along with the display of the relevant sequence of this promoter. The autoradiograph shows that in the wild type, under fully inducing (I) conditions, an extended protection region becomes evident that overlaps with the region covered by probe 1 and probe 2 in the band shift assays. The protected region contains the strictly conserved CGGAGA motif roughly in its centre and hypersensitive adenines (circled dots) appear in the flanking regions of this motif. This protection is not evident in the sample analysed from non-inducing (NI) conditions, indicating that the nuclear Yna1p-Yna2p heterodimer does not bind DNA in the absence of the inducer. In *YNA1Δ* and *YNA2Δ* strains incubated on nitrate (inducing conditions), the protected region is not present and this suggests that *in vivo* both proteins in the heterodimer are required to form this protection pattern. Due to this large protection window, we were not able to determine exactly which guanines and adenines are participating in the complex-DNA interaction. Besides the protected bases, we also noted the appearance of at least two hypersensitive adenines (indicated by circled dots) in the autoradiograph which were not present in the *in vitro* methylation control (indicated by V) in a region corresponding to the central part of probe 1 and the end of probe 2. It should be remarked that these hypersensitive bands always specifically appeared in both mutant strains when they were shifted to inducing conditions (nitrate). Generally, the appearance of such residues displaying hypersensitivity towards the methylating agent dimethylsulphate (DMS) is a good indication of local DNA structure distortions generated by binding events. The same principle would apply when Yna1p is binding alone. Note that *YNA1* is only expressed to a detectable level in a *YNA2Δ* strain under nitrate induction (Fig 1C, N condition; Fig 2C) and this fact would make it possible for the hypersensitive adenine to appear in the *YNA2Δ* mutant. Taken together, our *in vivo* footprint data suggest that a functional heterodimer of Yna1p-Yna2p binds to the region corresponding to probe 1 and probe 2 and that this binding depends on the presence of both activator proteins.

### Yna1p and Yna2p most efficiently bind the *YNT1* promoter as heterodimers but both are able to bind also as homodimers

**Fig 5:**
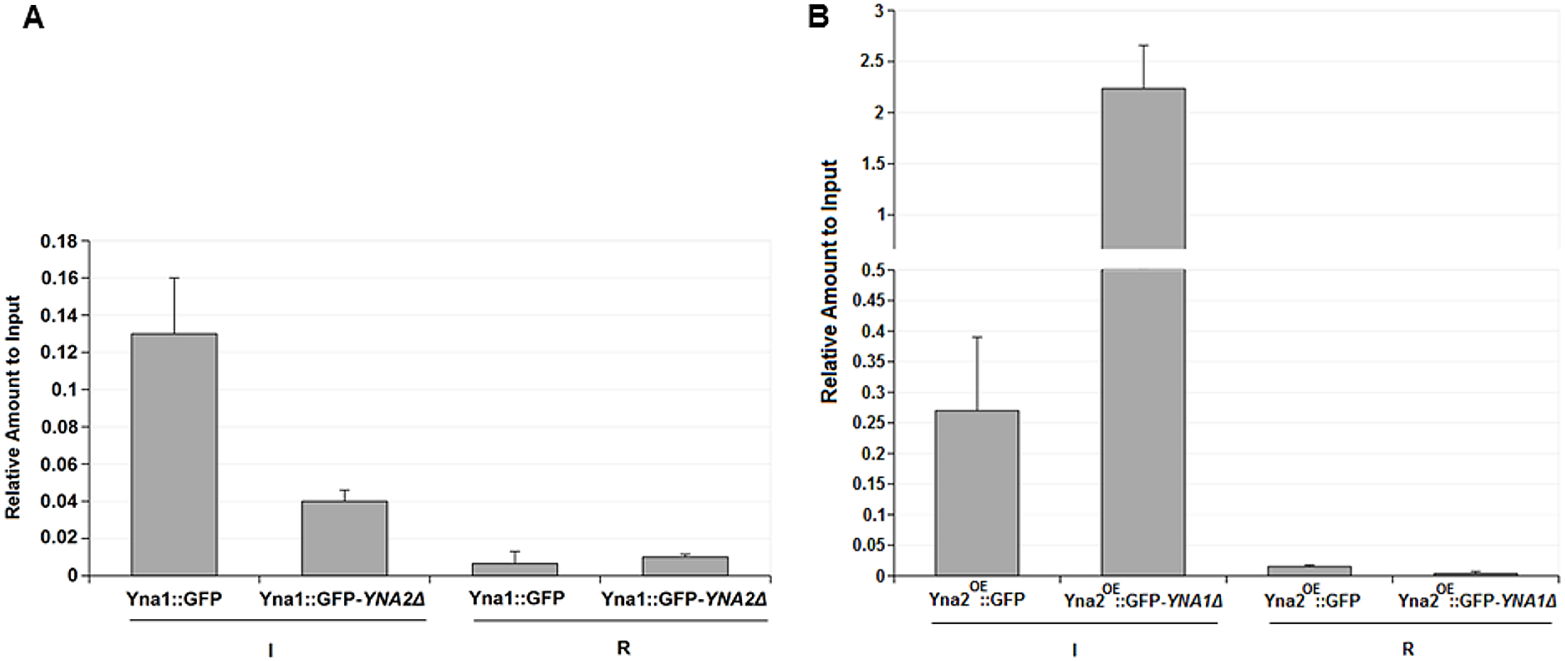
ChIP analysis of Yna1p and Yna2p occupying the *YNT1* gene promoter in different physiological conditions and genetic backgrounds. **A**. GFP-tagged Yna1p expressed from its own nitrate-inducible promoter in the wild type (Yna1::GFP) and *YNA2Δ* (Yna1::GFP-*YNA2Δ*) background was analysed by ChIP in cells grown under nitrate-inducing (I) or glutamine-repressing (R) conditions. **B**. Experimental set-up for the GFP-tagged Yna2p in the wild ytpe (Yna2^OE^::GFP) and *YNA1Δ* background (Yna2^OE^::GFP-*YNA1Δ*) was identical to the one described for Yna1p with the exception that the Yna2p-GFP fusion protein was expressed from the strong MOX promoter in order to be able to analyse Yna2p occupancy also in a *YNA1Δ* background in which *YNA2* is normally not expressed. All values shown in this figure represent the amount of *YNT1* promoter DNA relative to the amount of input DNA in the same sample. Experiments were done in two biological repetitions and in each experiment ChIP analysis was duplicated. Error bars thus represent the standard deviations of four measurements in each condition and strain.

We wished to analyse the DNA occupancy of Yna1p and Yna2p *in vivo* quantitatively by chromatin immunoprecipitation (ChIP) assays. To this end we performed the ChIP analysis on *H*. *polymorpha* cells grown under inducing (I, nitrate) and repressing (R, glutamine) conditions using the GFP-tagged versions of the activators as tag for IP (Fig 5).

The strains used for this analysis expressed the fusion proteins either in the wild type background (heterodimer possible, indicated in the figure as Yna1::GFP or Yna2::GFP) or in the mutant lacking the heterodimer partner protein (no heterodimers possible, indicated in the figure as Yna1::GFP-*YNA2Δ* or Yna2::GFP-*YNA1Δ*). The presence of the fusion proteins in the different backgrounds was analysed by qPCR targeting 114 nucleotides of the *YNT1* promoter region which contains the Yna1p/Yna2p 5′-CGGAGAATA-3′ binding motif. ChIP results are shown in Fig 5 and document that Yna1::GFP binds the *YNT1* promoter strongly only under inducing conditions (I), but can barely be detected under repressing conditions (Fig 5A). In agreement with *in vivo* footprinting ChIP results also show that high Yna1::GFP binding only occurs when Yna2p is present presumably allowing heterodimer formation. However, the fact that still around 30% of Yna1::GFP can be detected in the *YNA2Δ* background indicates that Yna1p can also bind to DNA as monomer or homodimer under induced conditions. A similar picture evolved when GFP-tagged Yna2p (indicated as Yna2^OE^::GFP) was tested in the ChIP assay. Also in this case, significantly more Yna2 protein is found associated with the *YNT1* promoter region under induced conditions compared to repressed conditions. But a striking feature of Yna2p binding was observed in the strain lacking Yna1p: in this case a roughly 10-fold higher amount of Yna2::GFP is precipitated compared to the amount detected in the wild type background. Strikingly, this hyper accumulation of Yna2::GFP is dependent on the presence of nitrate and does not occur on the repressing nitrogen source (glutamine). These data suggest that also Yna2p –similar to Yna1p –can bind as monomer or homodimer and needs the inducer for this activity. Obviously, in the absence of the heterodimer forming Yna1p, Yna2::GFP is present at higher amounts indicating that Yna1p balances the heterodimer formation with Yna2p under inducing conditions. The fact that Yna2 expression is driven by the strong *MOX* promoter cannot solely account for the hyper-accumulation of the GFP fusion protein as the amount of Yna2::GFP present at the *YNT1* promoter in the wild type background (putative heterodimer) is only around twice the amount of Yna1::GFP in the same heterodimeric complex (wild type background). In contrast, when Yna1p::GFP is lacking, the amount of Yna2p::GFP present as homodimer or monomer is roughly 10-fold higher than when Yna2p::GFP occupies the promoter in the heterodimeric Yna1p/Yna2p form.

### Chromatin remodelling in the *YNT1* promoter depends on both Yna1p and Yna2p

**Fig 6:**
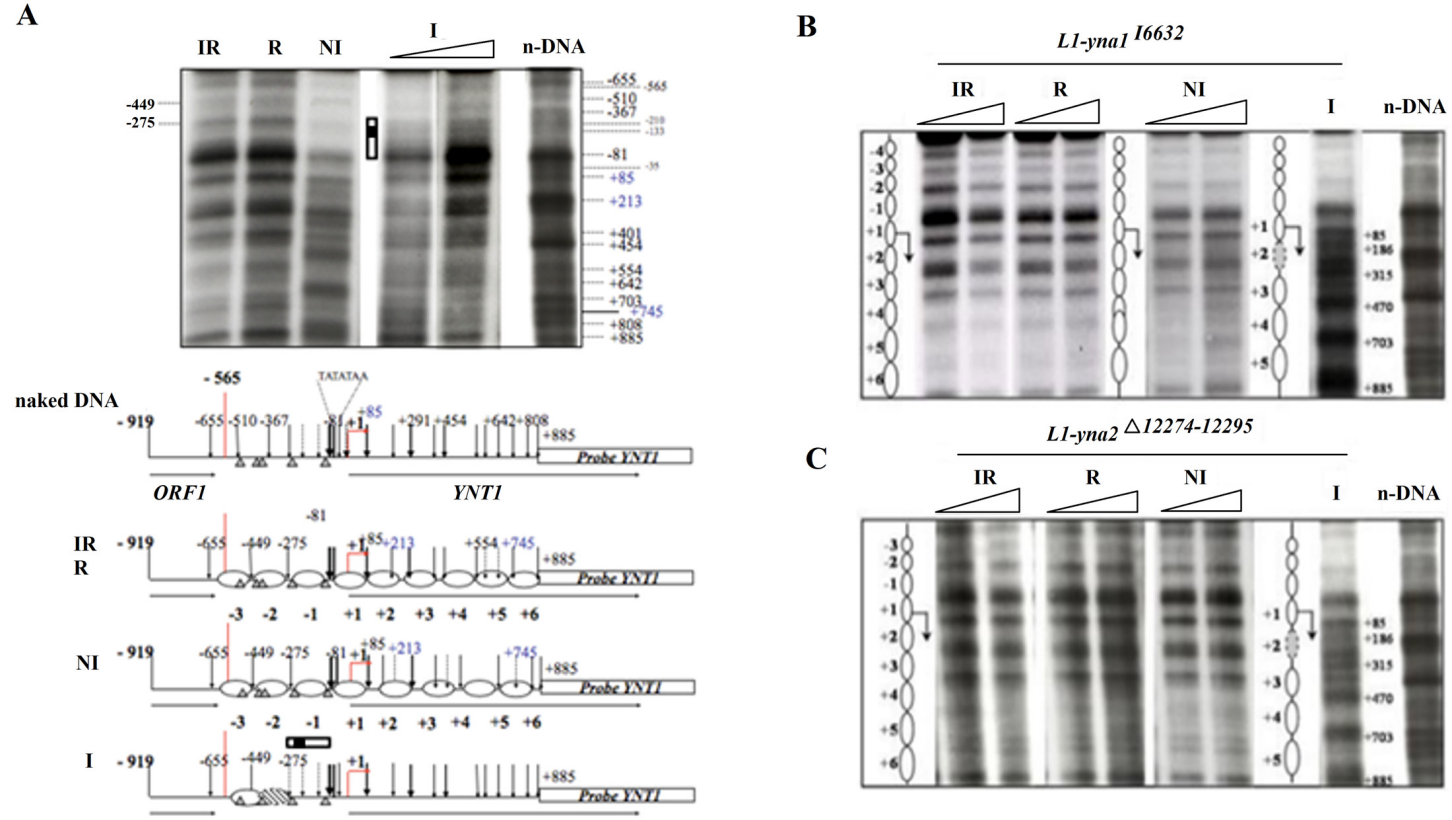
Chromatin remodelling in the *YNT1* gene promoter and ORF depends on both transcription factors. Autoradiographs are derived from MNase digestion and indirect-end labelling analysis of nucleosomes in the *YNT1* gene. Cells were incubated under the standard physiological conditions as indicated on top of the lanes (**I, nitrate induced, NI, non-induced on proline, IR, induced-repressed on nitrate plus glutamine and R, repressed on glutamine).** L1 wild type cells (**A**), L1-*yna1* ^*I6632*^ (**B**) and L1-*yna2* ^Δ*12274–12595*^ (**C**) were incubated under the indicated physiological conditions and subjected to chromatin analysis as detailed in Materials and methods. Nucleosomal pattern is compared to *in vitro* digested naked-DNA (n-DNA) Relevant sensitive site positions are indicated by arrows. IR, induction-repression; R, non-induction-repression; NI, non-induction-de-repression; I, induction-de-repression; n-DNA, naked DNA. In several cases two MNase concentrations (3,75 and 7,5 U/g of cells) were used for digestion, as represented by the white triangle above the lanes. The white rectangle, represents the dislocation site of nucleosome (-1) and the corresponding genomic region investigated by *in vivo* footprinting in Fig 4. The filled insert in this white rectangle represents the approximate position and size of the YNT1-probe 2 used in the EMSA assays. The nucleosomal distribution and MNase cutting sites on the *YNT1* promoter under the different physiological conditions is schematically represented. Ovals represent positioned nucleosomes, brindled ovals are partially positioned nucleosomes and vertical arrows indicate MNase cuts, their thickness being roughly proportional to site sensitivity. Putative GATA recognition sites for the wide-domain regulator NIT2 (GATA-sites) are indicated by triangles. +1 indicates the position of A in the ATG start codon, negative and positive numbers indicate nucleotide positions in the upstream and downstream region of the gene, respectively. TA-rich stretches are reported. Chromatin analysis in L1-*yna1* ^*I6632*^ and L1-*yna2* ^Δ*12274–12595*^ shows that nucleosomes maintain their position under all conditions.

As Yna1p and Yna2p directly bind to target promoters and initiate transcription, we determined if chromatin remodelling is also mediated by these two factors. To this end, we performed indirect end-labelling chromatin analysis following MNase digestion of intact *H*. *polymorpha* cells grown under different physiological conditions. As shown in Fig 6, there are drastic differences in the nucleosomal organisation in the promoter and the 5′ ORF region of the *YNT1* gene between different physiological conditions.

When the gene is silent under non-inducing (NI) and inducing-repressing (IR) conditions, three positioned nucleosomes are detectable in the promoter region (-3, -2, -1 upstream of the ORF and six positioned nucleosomes become evident inside the *YNT1* ORF (+1 to +6). Each of these nucleosomes is flanked by MNase sensitive sites located at positions +85, -81, -275, -449, -655. A very strong MNase hypersensitive site (indicated by a bold arrow in Fig 6A) becomes visible in chromatin isolated under conditions in which *YNT1* is not transcribed. In addition, there is a larger than expected distance (360 bp) between the MNase cutting sites corresponding to the end of nucleosomes -1 (position -275) and +1 (position + 85). Taken together, both these findings indicate that a nucleosome-free region may be extant between these nucleosomes potentially providing a docking platform for wide-domain and/or pathway-specific transcription factors. Upon nitrate induction a striking nucleosome rearrangement becomes detectable in which all nucleosomes in the transcribed region (+1–+6) as well two promoter nucleosomes (-1 and -2) lose their position. Chromatin restructuring in transcribed regions is known to be a consequence of transcription and thus it is not surprising that, when the gene is induced, nucleosomes starting from the transcriptional start point (nucleosome -1) and downstream lose their positioning. In many cases nucleosomes upstream of the transcriptional start point are remodelled by specific transcription factors and this process is independent from transcription [23]. To test this in *H*. *polymorpha*, we performed MNase assays in *YNA1Δ* and *YNA2Δ* strains and probed again the *YNT1* region for nucleosomal positioning. As seen in Fig 6 (panels B and C) nucleosomal rearrangements do not occur in either mutant. This is visible along the *YNT1* ORF and consistent with lack of gene transcription in both strains under inducing conditions. But also the upstream nucleosomes -1 and -2 do not rearrange in the mutants: this indicates that both regulators are involved in remodelling the chromatin in the promoter of this target gene. We also tested for chromatin structure changes for another gene belonging to the pathway. To this end, we targeted the nitrite reductase gene *YNI1* and analysed the MNase pattern obtained from cultures grown under conditions where the gene is silent (non-inducing, repressing, inducing-repressing) or activated (inducing conditions). This analysis is presented in S7 Fig and shows an identical picture to the *YNT1* pattern. Nucleosomes are strictly positioned under non-expressing conditions and lose this positioning upon gene induction. Remarkably, the nucleosome-free-region seems to be more extended in this gene than in *YNT1*. Nevertheless, a nucleosome-free region still extends here between nucleosomes -1 and + 1. As in *YNT1*, in *YNI1* all nucleosomal rearrangements are strictly dependent on the presence of both transcriptions factors (S7, S8 and S9 Figs).

## Discussion

Previous work has shown that in *H*. *polymorpha* the nitrate assimilation genes are regulated at the level of their cognate mRNA steady states, and most likely at the transcriptional level [5,6]. We have confirmed, refined and extended these findings, establishing that the appearance of the *YNT1*, *YNI1* and *YNR1* mRNAs follows that of the transcriptional activator genes *YNA1* and *YNA2*. The strong constitutive expression of *YNA2* suggests that this transcription factor is poised to initiate the regulatory process when nitrate becomes available. It is necessary for basic *YNA1* transcription which, through the nitrate-dependent positive autoregulation loop, subsequently further amplifies the induction signal leading to the formation and nuclear accumulation of a functional Yna1p-Yna2p heterodimer. While agreeing with the regulatory role proposed for Yna1p and Yna2p in previous studies [5,6], our experiments with the L1-*yna1* ^*I6632*^ and L1-*yna2* ^Δ12274–12595^ mutants disclose new features regarding the auto-regulatory role of Yna1p and Yna2p. In particular, while in Ávila (2002) no effect of the *YNA2* mutation on *YNA1* expression was reported, our experiments clearly reveal that, in the absence of Yna2p, *YNA1* expression is almost completely abolished under all conditions with the exception that it is weakly expressed on nitrate as sole nitrogen source. Considering that the *YNA1* gene promoter carries a potential binding site for the activators which may operate as heterodimer, this observation might be consistent with the proposed auto-regulatory function of the Yna1p-Yna2p heterodimer for the expression of the *YNA1* gene. Interestingly, *YNA2* is constitutively and strongly expressed independently of nitrate (although with a transient induction peak after 20 minutes on nitrate, see S1 Fig) and Yna1p appears to be required for *YNA2* expression. This suggests that Yna1p carries out both nitrate-dependent and nitrate-independent functions at the *YNA2* promoter. In other words, the functions of the two activators are inter-dependent and, specifically, a positive feedback loop exists in which Yna2p –constitutively present in the cell–facilitates *YNA1* gene expression. In turn, the latter acts as co-activator for *YNA2* transcription. Upon nitrate availability, this feedback loop would be enhanced and subsequently increase the level of the inducible Yna1p and of downstream targets of the activators, i.e. the structural genes necessary for assimilation of nitrate. We now know that the induction process of the considered nitrate-inducible gene responds only to a functional heterodimer of Yna1p and Yna2p. It is, however, surprising that also the expression of *YNA2* is dependent on *YNA1* because the *YNA2* gene is constitutively transcribed and it does not carry the CGGAGA box present in nitrate-responsive genes (*YNA1*, *YNI1*, *YNT1*, *YNR1*). This indicates that *YNA2* gene expression is not regulated by the Yna1-Yna2 heterodimer. Instead, Yna1p could bind as homo-dimer to a different sequence or may indirectly influence the *YNA2* expression, a situation that has been detected in our ChIP analysis of Yna1::GFP in the *YNA2* mutant (Fig 5).

The ChIP assay is able to quantify protein:DNA interactions and we could confirm our microscopic analysis of Yna1p::GFP and Yna2p::GFP subcellular localizations but also found that Yna1p can bind sequences in *YNT1* promoter in the absence of Yna2p. Plausibly, the binding must occur either as monomer or as homodimer, possibly interacting with different sequences than the heterodimer and providing an explanation for the finding that Yna1p is needed for *YNA2* expression although no classical “CGGAGA” motif is present in this promoter. The fact that much less Yna1::GFP is found at the *YNT1* promoter in the absence of Ynt2p is most likely due to a reduction in both nuclear accumulation and DNA-binding affinity of the monomer or homodimer. At the moment we can only speculate why less nuclear Yna1p is present in the absence of Yna2p but it is tempting to speculate that the permanently nuclear Yna2p is responsible for masking the NES of Yna1p thus preventing its interaction with the nuclear export machinery. However, this interpretation is only partially in agreement with the split-YFP interaction studies. They showed that the heterodimer between Yna1p and Yna2p does not form in the presence of glutamine (repressing conditions) but our microscopic studies clearly showed nuclear positioning of Yna1p::GFP under all conditions, including glutamine. This would mean that some other mechanism keeps Yna1p::GFP in the nucleus. Alternatively, Yna1p::YFP levels expressed from its own promoter might be insufficient for detection by fluorescence microscopy. This in fact seems to be the most likely explanation because *YNA1* is expressed to a very low level on glutamine (compare Fig 1B) and the reconstituted split-YFP is known to be less fluorescent than GFP.

On the other hand it is remarkable that in the absence of Yna1p around 10-fold more Yna2p are detected at the *YNT1* promoter compared to the wild type where Yna1p is present in the heterodimer. This surprising result suggests that the Yna2p monomers or homodimers strongly associate with DNA but we do not know if this binding is sequence-specific for the CGGAGA-box or not. In any case Yna1p seems to have an essential role in balancing the heterodimer to equimolar levels and prevent the hyper-accumulation of transcriptionally incompetent Yna2p mono- or homodimers at their common targets in nitrate-responsive genes.

To our surprise, this heterodimer is constitutively located to the nucleus and does not need the inducer to trigger nuclear accumulation. This is in contrast to the best studied fungal model system *A*. *nidulans* in which NirA accumulates in the nucleus only when nitrate is present inside the cell [52]. Similarly, also NLP7, a nitrate-responsive transcription factor in *Arabidopsis thaliana*, accumulates in the nucleus after nitrate induction [53]. It should be also noted that, the Yna1p up-regulation by nitrate and the formation of high amounts of a functional Yna1p-Yna2p heterodimer just under inducing conditions can also be seen as an indirect “nitrate-dependent nuclear accumulation”. This mechanism is functionally equivalent, but mechanistically different, to the situation extant in *A*. *nidulans* and *A*. *thaliana*. Many yeast transcription factors of the Zn(II)_2_Cys_6_ type (e.g. Gal4p, Put3p and Leu2p of *S*. *cerevisiae*) are also constitutively nuclear and bound to cognate UAS (Upstream Activation Sequences), but this does not automatically lead to target gene activation. Only upon dissociation of a repressing cofactor (e.g. GAL80p from GAL4p) the activation domain is exposed and target genes are expressed [19]. Only a few studies looking at this particular aspect of transcription factor regulation have been carried out in other fungi. As for *A*. *nidulans*, the proline activator PrnA has also a permanent nuclear localisation, but needs the inducer to bind to its DNA target sites *in vivo* [54]. Nevertheless, importantly, forced nuclear accumulation (blocking export) is not sufficient for NirA activity and this indicates a two-step process, where the nitrate not only blocks nuclear export of NirA, but also converts nuclear NirA from an inactive to an active configuration [55]. In *H*. *polymorpha* the Yna1p-Yna2p heterodimer shows constitutive nuclear localization. This implies that the presence of nitrate elicits heterodimer DNA-binding and/or a conformational activation of the DNA-bound proteins.

Yna2p is crucial for the nuclear import or retention of Yna1p. It is possible that the NLS of Yna1p is weakly active in the absence of Yna2p. Alternatively or additionally, Yna2p may mask the Yna1p-NES (“AKLTTLLNEMDDLRLD” [55]), thus preventing its export from the nucleus (see S5 Fig for the predicted NLS of these proteins).

We identified a common motif in the promoters of the Yna1p-Yna2p target genes. This nucleotide motif does not conform to the typical CCG-n_x_-CCG Zn(II)_2_Cys_6_ protein target site [20,56] but harbours the sequence 5′-CGGAGAATA-3′ (Fig 4A), similar to the structure of *A*. *nidulans* NirA binding site (5′-CTCCGHGG; H = A,C,T) [12]. In conclusion, we assume that the 9-nucleotide sequence represents the *H*. *polymorpha* UAS for the nitrate gene expression and the transcriptional activators Yna1p and Yna2p bind this site preferentially as a heterodimer.

We have also shown in this study that Yna1p and Yna2p physically interact and that this interaction is crucial for bringing the heterodimer to the nucleus or for keeping it there. Our *in vivo* footprinting results from the *YNT1* promoter region suggest, however, that this form of the transcription factor complex does not bind DNA in the absence of nitrate but needs the inducer to specifically interact with their recognition sequences in target genes. Our chromatin-immunoprecipitation studies confirmed these results by demonstrating quantitatively that *in vivo* both Yna1p and Yna2p only bind to the *YNT1* promoter when the inducer is present. Thus, although both proteins are present in the nucleus under all conditions, they still need the inducer to bind to their target promoters. At the moment it is not clear which biochemical changes occur upon nitrate induction that transform the inactive transcription factors to an active heterodimeric complex. A mechanism how a fungal nitrate regulator can respond to the presence of the inducer was recently discovered in *A*. *nidulans* [55]. In this paper we reported that reversal of methionine sulfoxidation in the nuclear export signal of NirA is associated with the transformation process. If a similar mechanism exists for *H*. *polymorpha*, is unknown.

This work also elucidates new aspects in the regulation of the *H*. *polymorpha* nitrate metabolism. Our data show the existence of hierarchies of repressing compounds, glutamine being the strongest repressing metabolite, and proline being a neutral, or nearly-neutral, nitrogen source. It has been proposed that the existence of such hierarchies is generally related to the conversion rate at which a given nutritional nitrogen compound is transformed into the effector molecules which respond to and signal the “nitrogen state” of the cell [57].

Taken the results of this study together we propose that the “CGGAGA” DNA motif present in the nitrate-responsive gene promoters serve as upstream-activating sequence (UAS) that mediate nitrate induction by serving as binding site for a functional Yna1p/Yna2p heterodimer. Formation of the heterodimer itself does not need the inducer, but stable binding of the heterodimeric activator complex to target DNA and transcriptional competence requires nitrate either as effector molecule or as signalling compound. However, the activator complex seems to be sensitive to glutamine repression because neither interaction nor transcriptional activity is detected under these conditions. UAS are conserved in promoters of all known genes involved in nitrate assimilation, including in the promoter of *YNA1*. This latter sequence arrangement results in a nitrate-responsive positive auto-regulation loop at the level of transcription factor gene expression and a balanced stoichiometry in the Yna1p/Yna2p heterodimer in which Yna2p is expressed to high levels constitutively and Yna1p is induced to high levels when the inducer and thus the potential substrate for the assimilation process becomes available. Although both proteins can bind individually, the balanced stoichiometry seems to be important for optimal function of the activator complex. To ensure optimal binding, binding sites of the regulators in the investigated target gene promoters appear to lie preferentially in a nucleosome-free region that presumably allows efficient DNA recognition without nucleosomal barriers. Ultimately these binding events lead to a drastic chromatin rearrangement, followed by the transcriptional activation process. The latter is likely mediated through the recruitment of remodelling complexes, presumably containing histone acetyl-transferases and/or ATP-dependent remodelling factors along with the pre-initiation complex.

## Supporting Information

S1 Fig Northern blots showing the time course of induction on the nitrate cluster gene mRNA levels. *H*. *polymorpha* L1 cells, pre-grown in proline, were incubated in nitrate for the indicated time period. Numbers below the individual lanes represent relative expression values calculated from densitometric measurements of the autoradiograph, normalized to the loading control signal (actin gene *HpACT*).
(TIF)

S2 Fig Yna1 null mutant. **A.** Schematic representation of the *YNA1 locus* and adjacent regions and of the LD-I cassette used to generate the *yna1* ^*I6632*^::*LEU2* allele (EV, *EcoRV*; S, *SacI*; EI, *EcoRI*). Probe-Y1, used for Southern analyses is also indicated (panel C). **B.** Growth tests show that L1- *yna1* ^*I6632*^ is unable to used nitrate as sole nitrogen source for growth. L1+p37 (Leu^+^) is the wt strain with a *LEU2* containing vector (EBOX37p6); yna1Δ is the L1*-yna1^I6632^* mutant; yna1Δ+pA1 is the same mutant with a *YNA1* containing plasmid; yna1Δ−pA1 is a plasmid-less derivative of the previous strain. **C.** Southern blot showing the *yna1^I6632^* null allele. Genomic DNA, digested with *EcoRV*, was hybridized with probe-Y1 (panel A). Lane 1, wt (L1); lane 2, null mutant (L1*- yna1* ^*I6632*^)
(TIF)

S3 Fig Yna2 null mutant. **A.** Schematic representation of the *YNA2 locus* and adjacent regions and of the strategy used to generate the *yna2* ^Δ*12274–12595*^:: *LEU2* allele (P, *Pst*I; EV, *Eco*RV; C, *Cla*I). Probe-*Y2*, used for Southern analyses is also indicated (panel C). **B.** Growth tests showi that L1- *yna2* ^Δ*12274–12595*^ is unable to use nitrate as sole nitrogen source for growth. L1+p42 (Leu+) is the wild type with a *LEU2* containing vector (EBOX42p1); *yna2*Δ is the L1- *yna2* ^Δ12274–12595^ mutant; *yna2*Δ+pA2 is the same mutant with a *YNA2* containing plasmid; *yna2*Δ−pA2 is a plasmid- less derivative of the previous strain. **C.** Southern blot showing the *yna2* ^*Δ12274–12595*^ null allele. Genomic DNA, digested with *Pst*I, was hybridized with probe-*Y2* (panel A). Lane 1, wt (L1); lane 2, null mutant (L1- *yna2* ^Δ*12274–12595*^)
(TIF)

S4 Fig Nuclear accumulation of Yna1p is independent from nitrate and Yna2p-dependent also on other nitrogen sources. Yna1p expressed as a GFP fusion from its own promoter (left panel) and Yna2p-GFP expressed from the *MOX* promoter (right panel) was analyzed by confocal microscopy in the wild type (L1) strain and in strains lacking *YNA1* (L1-*yna1* ^*I6632*^) or *YNA2* (L1-*yna2* ^*Δ12274–12595*^) under non-inducing (NI, proline), inducing-repressing (IR, nitrate and glutamine) or repressing (R, glutamine) conditions. Below the recorded fluorescent images the corresponding DIC image is shown. Scale bars refer to 2 μm.
(TIF)

S5 Fig ClustalW alignment of the pathway-specific transcription factors in *N*. *crassa* (NIT4), *A*. *nidulans* (NIRA) and *H*. *polymorpha* (Yna1p and Yna2p). The sequences aligned comprise the DNA binding domain (DBD) showing the six conserved cysteines, the putative nuclear localization signals (NLS1 and NLS2) and the dimerization domain characterized by the presence of regularily spaced leucines and confirmed so far to be functionally required for in vitro DNA binding of NirA in *A*.*nidulans*.
(TIF)

S6 Fig Purified fractions of Yna1p(1–185) and Yna2p(1–180) expressed in *E*. *coli*. I, input; W, protein composition from the column first wash; 1, first elution fraction; 2, second elution fraction; 3, third elution fraction; 4, fourth elution fraction; 5, fifth elution fraction. Fractions 3 are used for band shift assays shown in Fig 4.
(TIF)

S7 Fig Nucleosomal organization of the *YNI1* promoter region. **A.** Location of the analyzed region in the context of the nitrate utilization cluster. The *EcoRI-EcoRV* fragment used as probe is also shown. **B.** Representative autoradiograms showing the MNase digestion patterns of the analyzed region. Relevant MNAse hypersensitive sites are indicated. IR, induced-repressed conditions (nitrate + glutamine); R, repressed conditions (glutamine); NI, non-induced conditions (proline); I, induced conditions (nitrate); n-DNA, naked DNA. Two MNase concentrations were used for each condition, as represented by the horizontal triangles above the lanes (3,75 and 7,5 U/g of cells). See Materials and Methods for technical details. **C.** Schematic representation of the chromatin organization of the *YNI1* promoter region in the physiological conditions examined. Ovals indicate positioned nucleosomes, vertical arrows indicate MNase cutting sites, their thickness being roughly proportional to site sensitivity. *YNI1* translational start is indicated by an horizontal arrow at position +1, as well as the *YNT1* end (dashed vertical line; -211). Putative GATA recognition sites are indicated by grey triangles at positions -123 and -162.
(TIF)

S8 Fig Expression and chromatin structure of *YNI1* in the wild type and mutants. Expression of the *YNI1* gene and chromatin structure in the promoter established in the wild type strain and in the regulatory mutants. All growth conditions were as in as in S7 Fig with one additional “induced” condition in which nitrate was added simultaneously with the neutral nitrogen source proline (Ipro). Strains were identical to the ones used in S7 Fig and L1 indicates wild type, Δ1 indicates L1-*yna1* ^*I6632*^, and Δ2 L1- *yna2* ^Δ*12274–12595*^. The RNAs were prepared from the same cultures used for chromatin analysis. **A.** Northern blots. **B.** MNase digests. The chromatin organization of the wild type (L1 strain) is schematized to the left of each panel, that of the mutants to the right of each panel. Arrows, translational start. Grey triangles, nucleosome free regions (nfr). Dots, hypersensitive sites present in L1- *yna2* ^Δ*12274–12595*^ (Δ2) under R and RI conditions.
(TIF)

S9 Fig Northern blots and MNase digests at 20 min after induction. *H*. *polymorpha* L1, L1- *yna1* ^*I6632*^ (Δ1) and L1- *yna2* ^Δ*12274–12595*^ (Δ2), grown under I conditions were examined. Results for the L1 strain at 120 min are also shown to allow comparisons to the standard conditions. Total RNAs were prepared from the same cultures used for chromatin analysis (A) Northern blots (B) MNase digestion pattern and nucleosomal organization patterns. The chromatin organization is schematized to the left of each of the 20 min panels. For the L1, 120 min panel, to the left, chromatin structure under induced (I) conditions, to the right chromatin structure under non induced (NI) condition. Dots indicate two MNase sensitive sites present in the wt after 20 min induction (positions -618 and -509) which are absent after 2 hours induction.
(TIF)

S1 Table Oligonucleotides used in this work. (DOCX)
