# Supplementary figures and images for: Interaction of Yna1 and Yna2 Is Required for Nuclear Accumulation and Transcriptional Activation of the Nitrate Assimilation Pathway in the Yeast Hansenula polymorpha
Source: PLoS One. 2015 Sep 3;10(9):e0135416. doi: 10.1371/journal.pone.0135416 (PMC4559421; doi:10.1371/journal.pone.0135416)

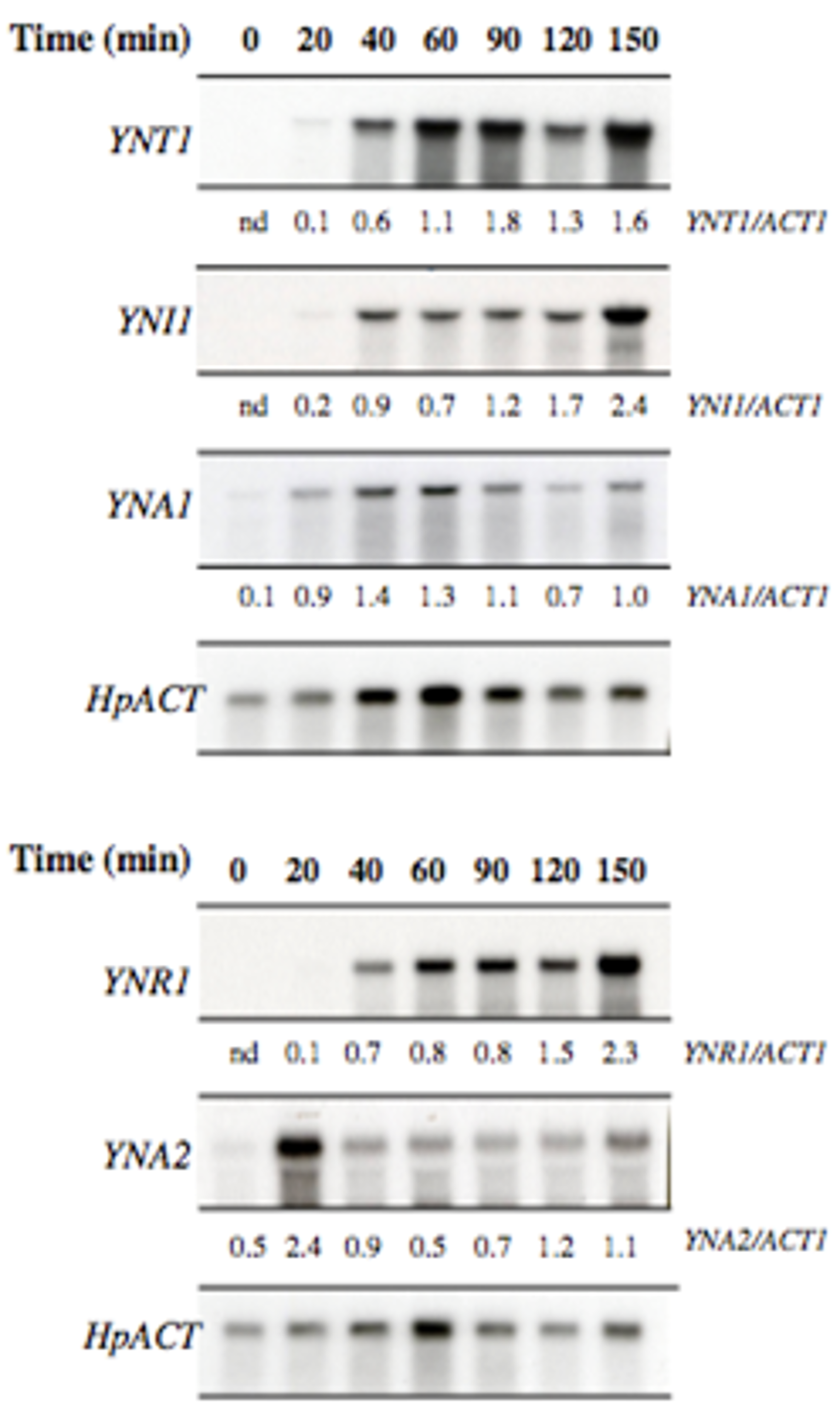

Supplement: S1 Fig — H. polymorpha L1 cells, pre-grown in proline, were incubated in nitrate for the indicated time period. Numbers below the individual lanes represent relative expression values calculated from densitometric measurements of the autoradiograph, normalized to the loading control signal (actin gene HpACT). (TIF) [file pone.0135416.s001.tif]

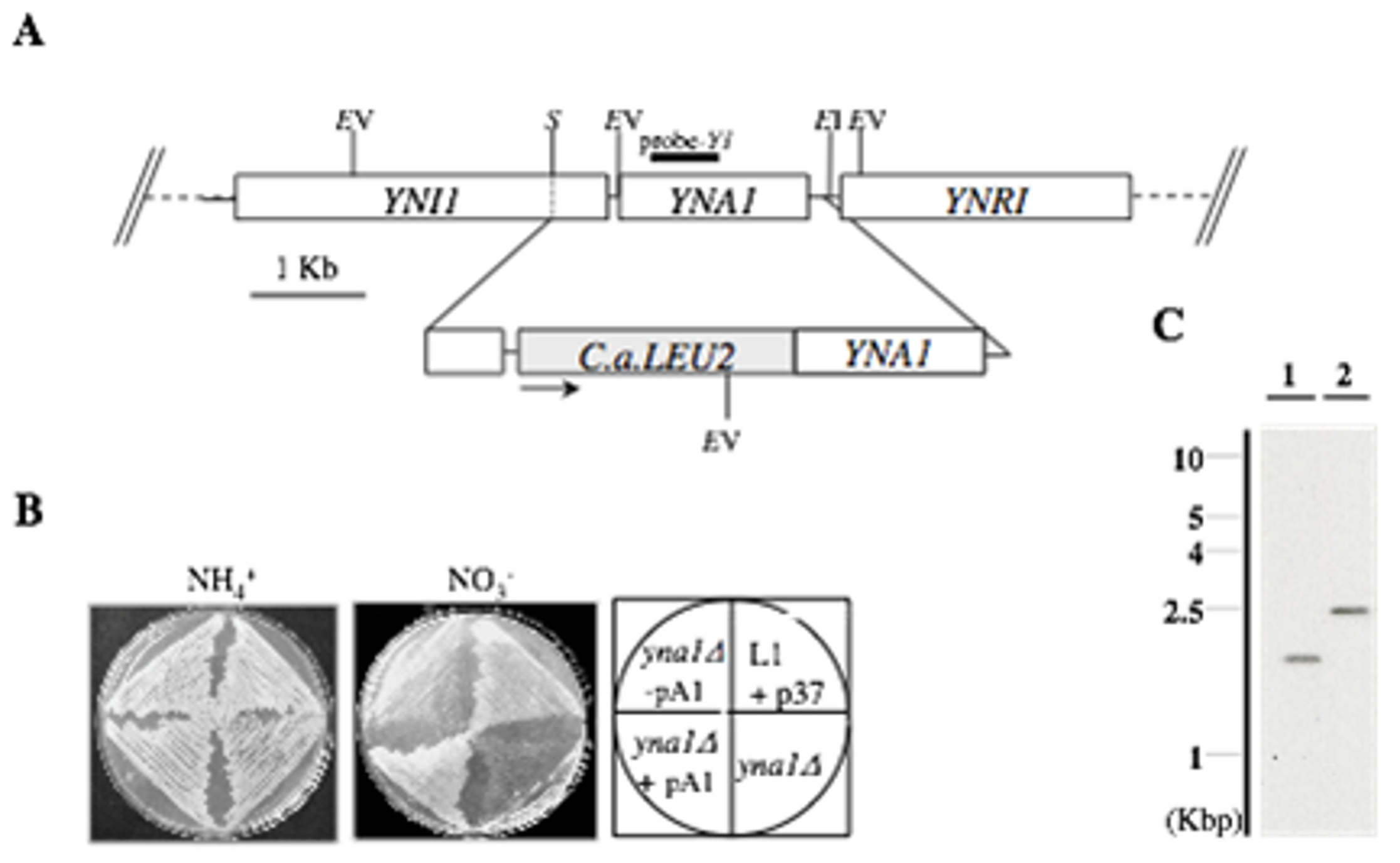

Supplement: S2 Fig — A. Schematic representation of the YNA1 locus and adjacent regions and of the LD-I cassette used to generate the yna1 I6632::LEU2 allele (EV, EcoRV; S, SacI; EI, EcoRI). Probe-Y1, used for Southern analyses is also indicated (panel C). B. Growth tests show that L1- yna1 I6632 is unable to used nitrate as sole nitrogen source for growth. L1+p37 (Leu+) is the wt strain with a LEU2 containing vector (EBOX37p6); yna1Δ is the L1-yna1I6632 mutant; yna1Δ+pA1 is the same mutant with a YNA1 containing plasmid; yna1Δ−pA1 is a plasmid-less derivative of the previous strain. C. Southern blot showing the yna1I6632 null allele. Genomic DNA, digested with EcoRV, was hybridized with probe-Y1 (panel A). Lane 1, wt (L1); lane 2, null mutant (L1- yna1 I6632) (TIF) [file pone.0135416.s002.tif]

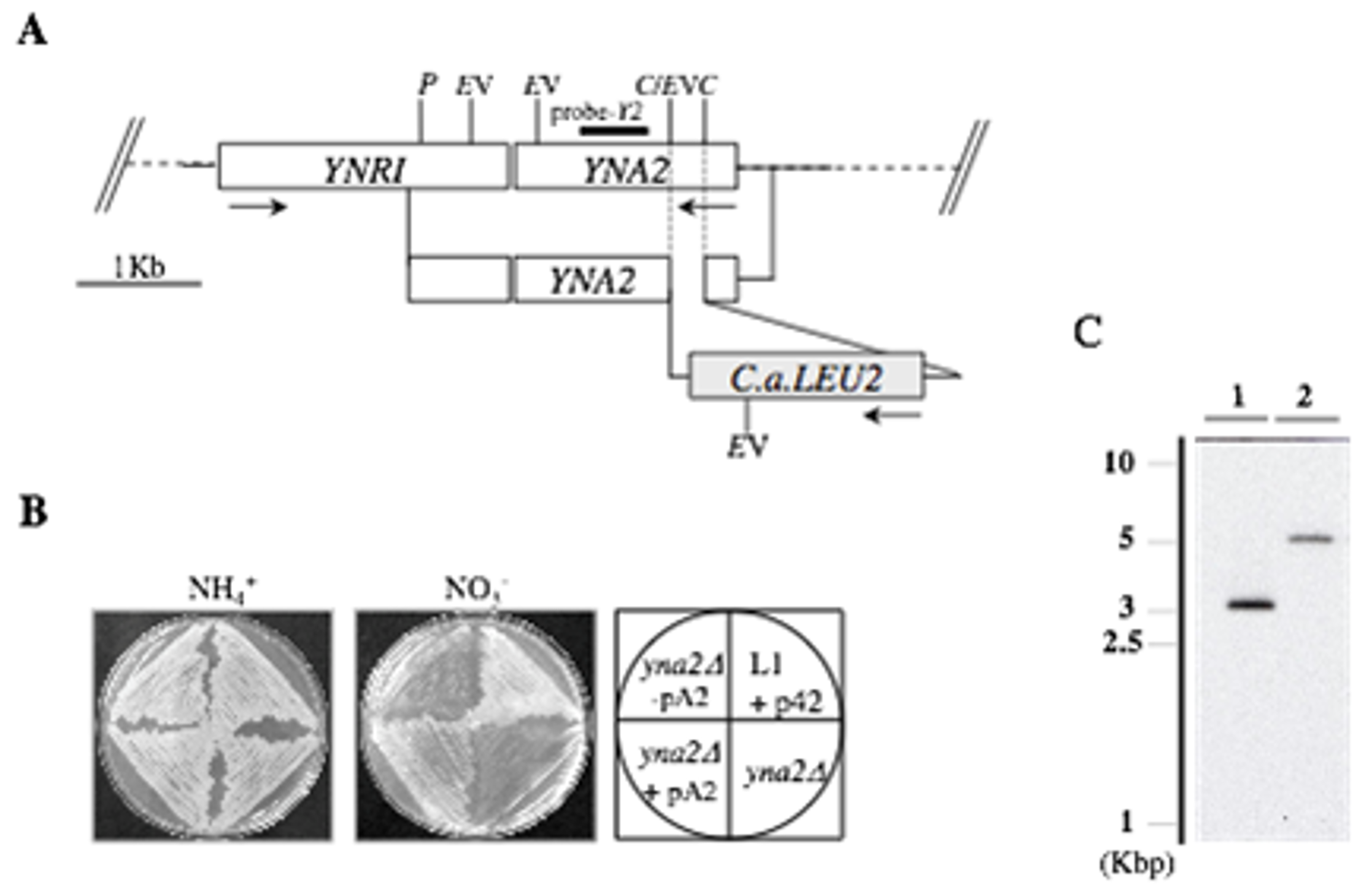

Supplement: S3 Fig — A. Schematic representation of the YNA2 locus and adjacent regions and of the strategy used to generate the yna2 Δ12274–12595:: LEU2 allele (P, PstI; EV, EcoRV; C, ClaI). Probe-Y2, used for Southern analyses is also indicated (panel C). B. Growth tests showi that L1- yna2 Δ12274–12595 is unable to use nitrate as sole nitrogen source for growth. L1+p42 (Leu+) is the wild type with a LEU2 containing vector (EBOX42p1); yna2Δ is the L1- yna2 Δ12274–12595 mutant; yna2Δ+pA2 is the same mutant with a YNA2 containing plasmid; yna2Δ−pA2 is a plasmid- less derivative of the previous strain. C. Southern blot showing the yna2 Δ12274–12595 null allele. Genomic DNA, digested with PstI, was hybridized with probe-Y2 (panel A). Lane 1, wt (L1); lane 2, null mutant (L1- yna2 Δ12274–12595) (TIF) [file pone.0135416.s003.tif]

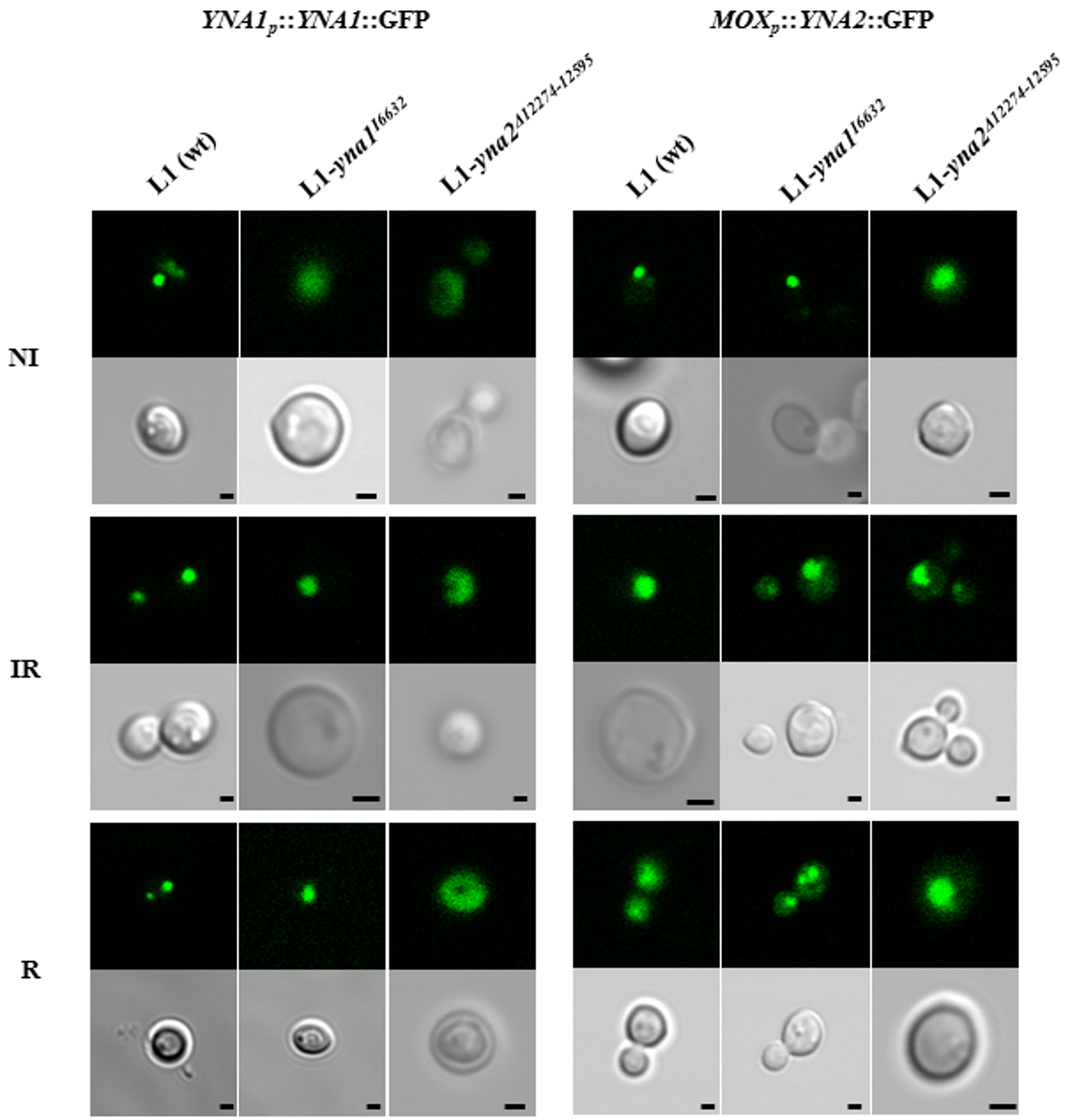

Supplement: S4 Fig — Yna1p expressed as a GFP fusion from its own promoter (left panel) and Yna2p-GFP expressed from the MOX promoter (right panel) was analyzed by confocal microscopy in the wild type (L1) strain and in strains lacking YNA1 (L1-yna1 I6632) or YNA2 (L1-yna2 Δ12274–12595) under non-inducing (NI, proline), inducing-repressing (IR, nitrate and glutamine) or repressing (R, glutamine) conditions. Below the recorded fluorescent images the corresponding DIC image is shown. Scale bars refer to 2 μm. (TIF) [file pone.0135416.s004.tif]

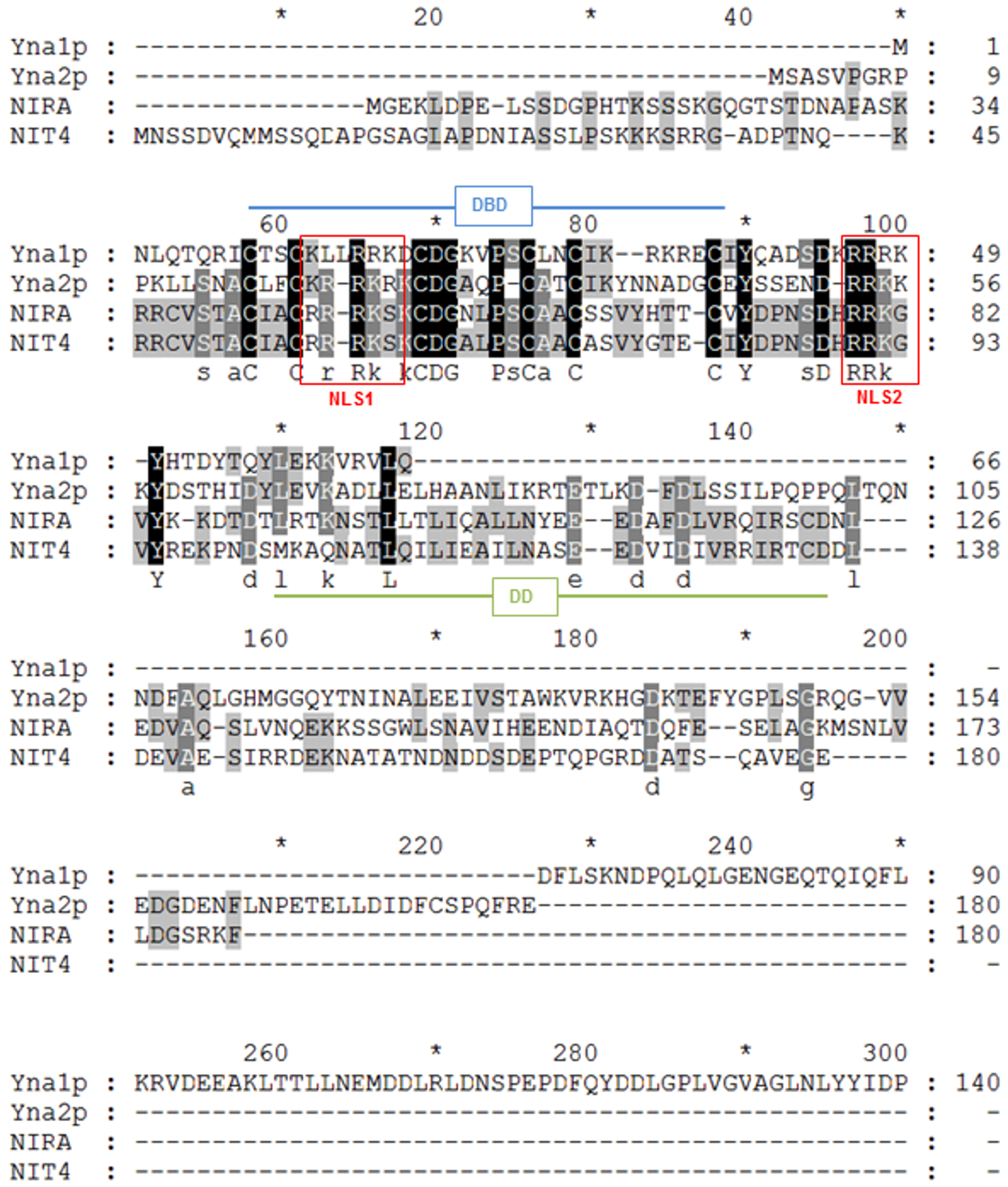

Supplement: S5 Fig — The sequences aligned comprise the DNA binding domain (DBD) showing the six conserved cysteines, the putative nuclear localization signals (NLS1 and NLS2) and the dimerization domain characterized by the presence of regularily spaced leucines and confirmed so far to be functionally required for in vitro DNA binding of NirA in A.nidulans. (TIF) [file pone.0135416.s005.tif]

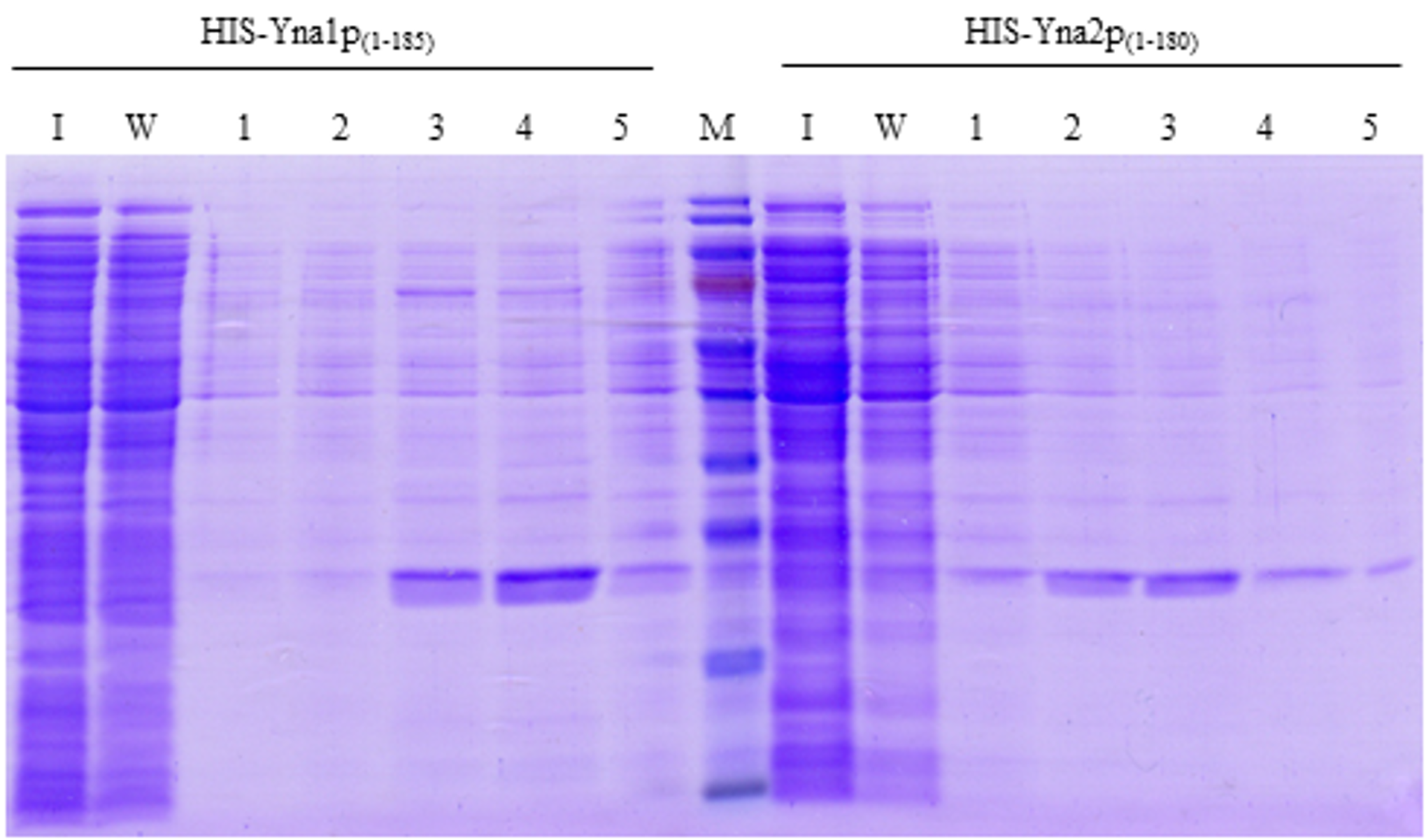

Supplement: S6 Fig — I, input; W, protein composition from the column first wash; 1, first elution fraction; 2, second elution fraction; 3, third elution fraction; 4, fourth elution fraction; 5, fifth elution fraction. Fractions 3 are used for band shift assays shown in Fig 4. (TIF) [file pone.0135416.s006.tif]

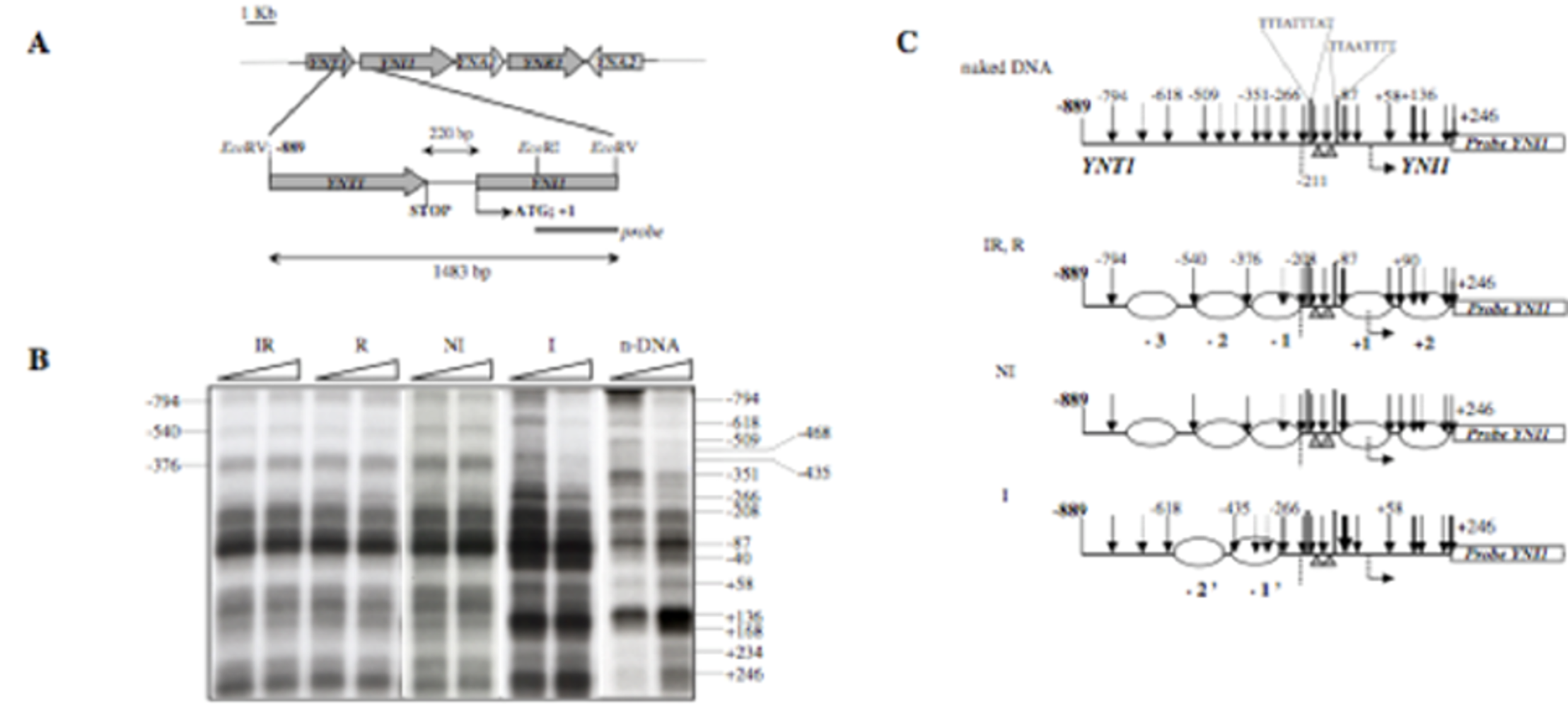

Supplement: S7 Fig — A. Location of the analyzed region in the context of the nitrate utilization cluster. The EcoRI-EcoRV fragment used as probe is also shown. B. Representative autoradiograms showing the MNase digestion patterns of the analyzed region. Relevant MNAse hypersensitive sites are indicated. IR, induced-repressed conditions (nitrate + glutamine); R, repressed conditions (glutamine); NI, non-induced conditions (proline); I, induced conditions (nitrate); n-DNA, naked DNA. Two MNase concentrations were used for each condition, as represented by the horizontal triangles above the lanes (3,75 and 7,5 U/g of cells). See Materials and Methods for technical details. C. Schematic representation of the chromatin organization of the YNI1 promoter region in the physiological conditions examined. Ovals indicate positioned nucleosomes, vertical arrows indicate MNase cutting sites, their thickness being roughly proportional to site sensitivity. YNI1 translational start is indicated by an horizontal arrow at position +1, as well as the YNT1 end (dashed vertical line; -211). Putative GATA recognition sites are indicated by grey triangles at positions -123 and -162. (TIF) [file pone.0135416.s007.tif]

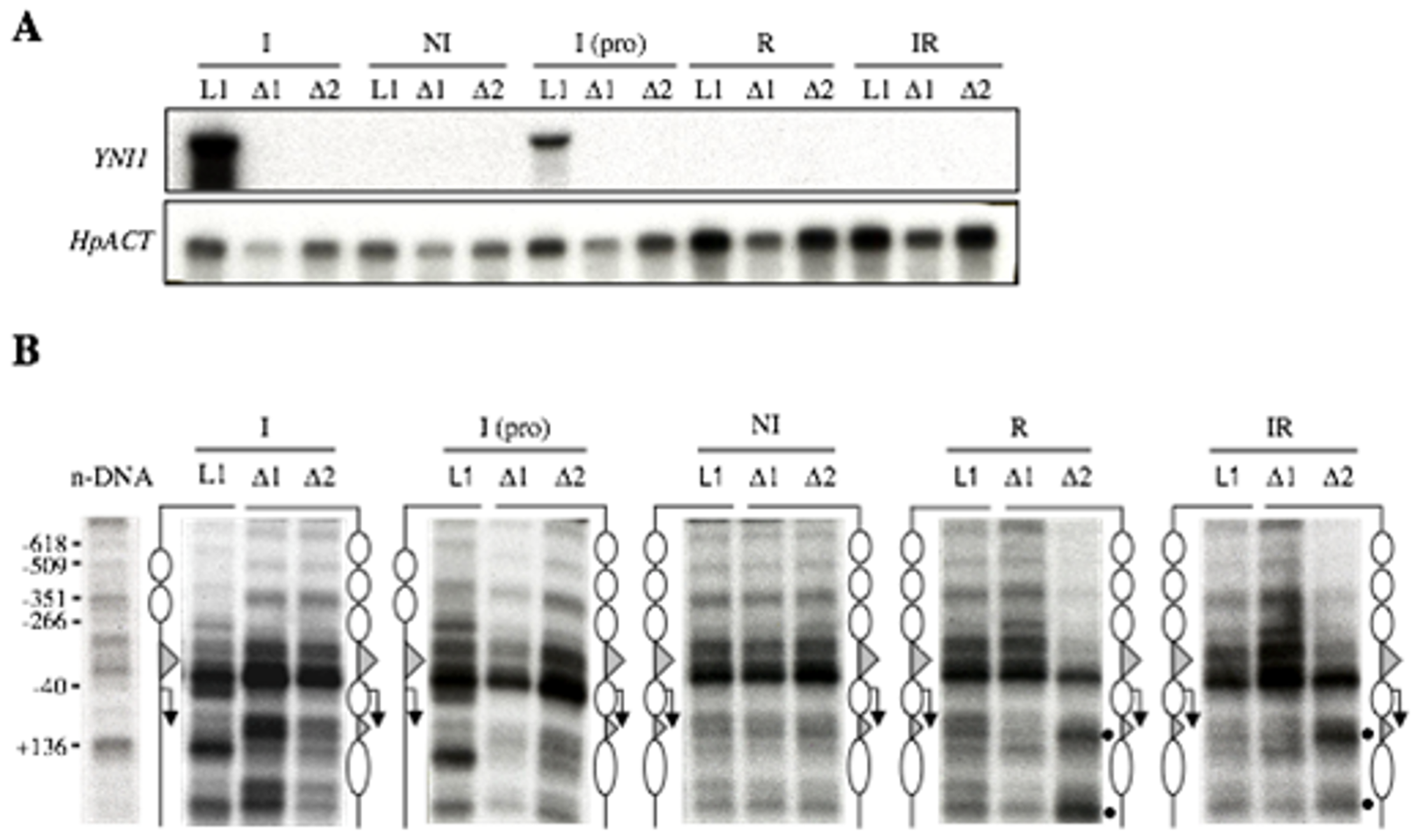

Supplement: S8 Fig — Expression of the YNI1 gene and chromatin structure in the promoter established in the wild type strain and in the regulatory mutants. All growth conditions were as in as in S7 Fig with one additional “induced” condition in which nitrate was added simultaneously with the neutral nitrogen source proline (Ipro). Strains were identical to the ones used in S7 Fig and L1 indicates wild type, Δ1 indicates L1-yna1 I6632, and Δ2 L1- yna2 Δ12274–12595. The RNAs were prepared from the same cultures used for chromatin analysis. A. Northern blots. B. MNase digests. The chromatin organization of the wild type (L1 strain) is schematized to the left of each panel, that of the mutants to the right of each panel. Arrows, translational start. Grey triangles, nucleosome free regions (nfr). Dots, hypersensitive sites present in L1- yna2 Δ12274–12595 (Δ2) under R and RI conditions. (TIF) [file pone.0135416.s008.tif]

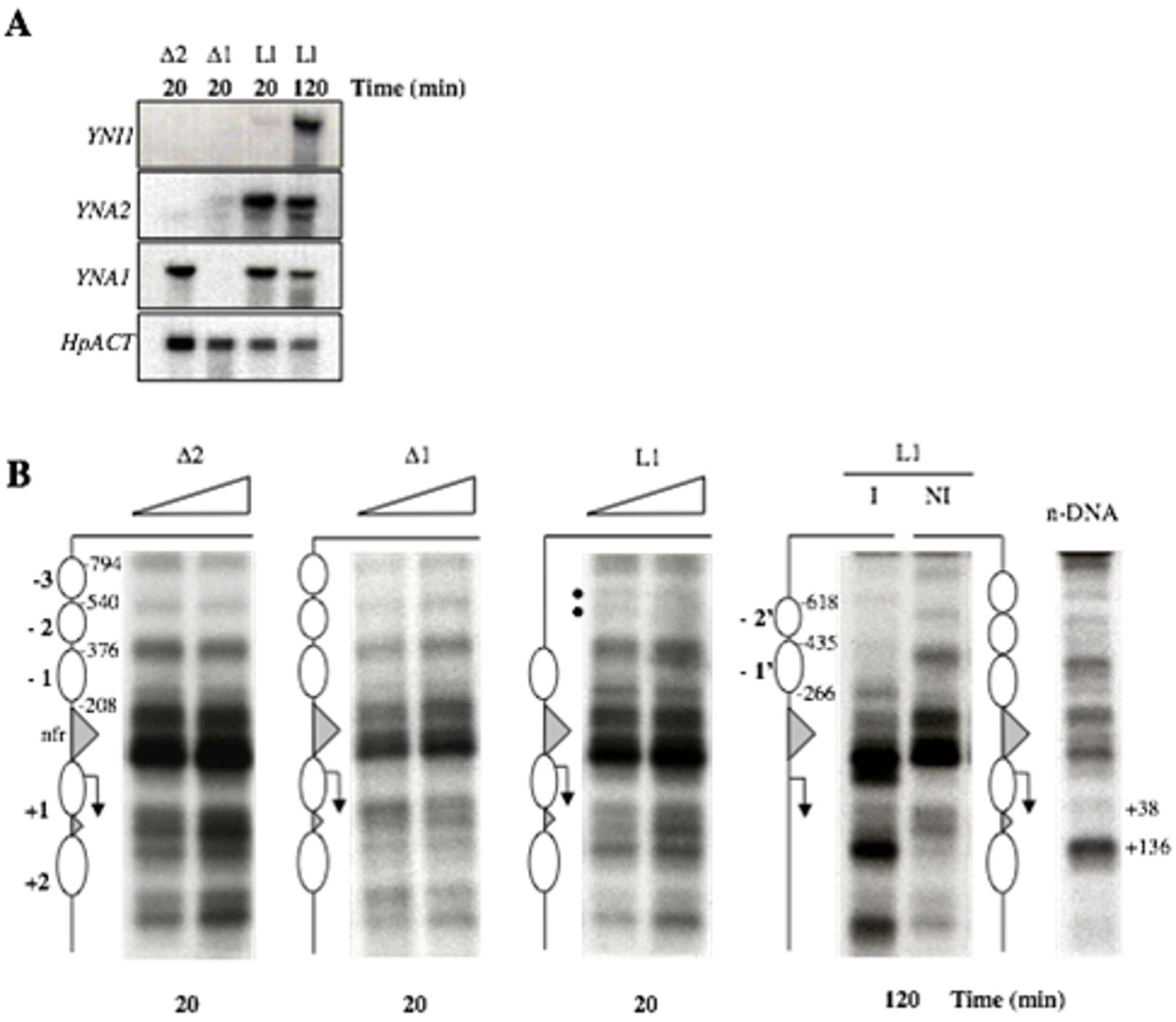

Supplement: S9 Fig — H. polymorpha L1, L1- yna1 I6632 (Δ1) and L1- yna2 Δ12274–12595 (Δ2), grown under I conditions were examined. Results for the L1 strain at 120 min are also shown to allow comparisons to the standard conditions. Total RNAs were prepared from the same cultures used for chromatin analysis (A) Northern blots (B) MNase digestion pattern and nucleosomal organization patterns. The chromatin organization is schematized to the left of each of the 20 min panels. For the L1, 120 min panel, to the left, chromatin structure under induced (I) conditions, to the right chromatin structure under non induced (NI) condition. Dots indicate two MNase sensitive sites present in the wt after 20 min induction (positions -618 and -509) which are absent after 2 hours induction. (TIF) [file pone.0135416.s009.tif]
