# Supplementary material for: Interaction of Yna1 and Yna2 Is Required for Nuclear Accumulation and Transcriptional Activation of the Nitrate Assimilation Pathway in the Yeast Hansenula polymorpha
Source: PLoS One. 2015 Sep 3;10(9):e0135416. doi: 10.1371/journal.pone.0135416 (PMC4559421; doi:10.1371/journal.pone.0135416)
Supplement: S1 Table — (DOCX) [file pone.0135416.s010.docx]

|  | |  | | |  |
| --- | --- | --- | --- | --- | --- |
| Name | |  | Nucleotide sequence |  |  |
|  |  |  | | |  |
| GATYNA1_fw | | 5’-GGGGACAAGTTTGTACAAAAAGCAGGCTT CATGAACTTGCAGACCCAGAGGATCTGC-3’ | | |  |
| GATYNA1tr_rv | | 5’-GGGA CCACTTT GTACAA GAAA GCT GGGT CTT ACCATAAGCAGCT GAT GGGAA -3’ | | |  |
| GATYNA2_fw | | 5’-GGGGA CAA GTTT GTACAAAAAAGCA GGCT TCATGTCCGCTT CCGT CCCT GGA -3’ | | |  |
| GATYNA2tr_fw | | 5’-GGGGA CCACTTT GTA CAA GAAAGCT GGG TCTTACTCTCT GAACT GT GGT GA GCA -3’ | | |  |
| YNA1gfp_fw | | 5’-GCGGCCGCGT GCCTTT GT GCTAAACCA G-3’ | | |  |
| YNA1gfp_rv | | 5’-CCCGGGAAATTTT GT CCA CCT GGA CG-3’ | | |  |
| BamYNA2gfp_fw | | 5’-ACCCCGGGA GCAAAATCGAAATCGAAAC-3’ | | |  |
| YNA2dr | | 5’-ATAGGGAT CCTT CATCAT GT CCGCT TCCGT -3’ | | |  |
| CCYNA1F | | 5’-ACGAGCT CTATT GCT CT CA G-3’ | | |  |
| CCYNA1R | | 5’-ACCTGGA CGA GAACGTTT C-3’ | | |  |
| YNA2df | | 5’-ATAGGGAT CCCCAACTTACCA GGTT CT GCA-3’ | | |  |
| YNA2er | | 5’-ATAGGGAT CCGGGT T CCT GGCACT TCTTTA -3’ | | |  |
| YNA1df | | 5’-TACTGTCGACAT GA GAT CA GGT GGT CTT CG-3’ | | |  |
| YNA1dr | | 5’-ACGCGAATT CA GAT CT GTT GGAA GCACCT C -3’ | | |  |
| leu2cf | | 5’-ATCCCCCGGGT GGGTTT GGT GAA GCA GCAA -3’ | | |  |
| leu2cr | | 5’-ATCCCCCGGGCGAATTT GGAAACAA GCCCG-3’ | | |  |
| YNA1SF | | 5’-ATGGT CCCTT CGT GGA GATA -3’ | | |  |
| YNA1SR | | 5’-GCCA GT GAA GAT CATCCA GA -3’ | | |  |
| s out2F | | 5’-AACATGGCACA CT GGAA GA G-3’ | | | |
| s out2R | | 5’-CATGGCCCAA GA CT CAA GTT-3’ | | | |
| YNT1SF | | 5’-ATGGT CCCTT CGT GGA GATA -3’ | | | |
| YNT1SR | | 5’-GCCA GT GAA GAT CATCCA GA -3’ | | | |
| YN2SF | | 5’-AAGCAGCCAAA GCT CT CTA C-3’ | | | |
| YN1SR | | 5’-ACATCGACACCCAT CA GCTT -3’ | | | |
| YNRSF | | 5’-AGGCA GA GGGT GGA GATATT-3’ | | | |
| YNR1SR | | 5’-TGTTCT GAAT GCT CCA GGT C-3’ | | | |
| YNA2ST | | 5’-AACTTGAGT CTT GGGCCAT C-3’ | | | |
| YNA2SR | | 5’-TTGCTCA GCTA GGACA CAT G-3’ | | | |
| ACTes a | | 5’-TGACT GA GGCTCCAAT GAA C-3’ | | | |
| ACT1HPF | | 5’-CCATCAGGCAACT CATA GGA -3’ | | | |
| YNT1 probe1 | | 5’-CCCGCT CAT CGGA GAATATTCT GCT CAAAAT TAAGGATGCCCAT-3’ | | | |
| YNT1 probe 2 | | 5´-GGCTA GCAT CCCGCT CAT CGGA GAATATT CT GC-3´ | | | |
| m1 | | 5´-GGCTA GCAT CAATCT CAT CGGA GAATATT CT GC-3´ | | | |
| m2 | | 5´-GGCTA GCAT CCCGCT CATATTA GAATATTCT GC-3´ | | | |
| Primer1_s ens e | | 5’-TCTTCAAGA GA GAAATCCGAA GTA GA CG-3' | | | |
| Primer2_s ens e | | 5'-ACCTCACGTT GGA GCGGTA CAAAAATGG-3' | | | |
| Primer3_s ens e | | 5'-GCGGTA CAAAAATGGGCGCACTTA CAT GT G-3' | | | |
| primer linker long | | 5’-GCGGT GA CCCGGGA GATCT GAATT C-3’ | | | |
| primer linker s hort | | 5’-GAATTCAGAT C-3’ | | | |
| YT_Ch_fw | | 5´-AAATGGGCGCACTTACATGTGATAGTG-3 | | | |
| YT_Ch_rv | | 5´-ATACCTATTTCCGTAATGGGCATCCTTAATT-3 | | | |

**S1 Table**. Primers used in this work
